# Highly conserved motifs in non-coding regions of Sirevirus retrotransposons: the key for their pattern of distribution within and across plants?

**DOI:** 10.1186/1471-2164-11-89

**Published:** 2010-02-04

**Authors:** Alexandros Bousios, Nikos Darzentas, Athanasios Tsaftaris, Stephen R Pearce

**Affiliations:** 1 Department of Biology and Environmental Science, School of Life Sciences, University of Sussex, Brighton, UK; 2 Institute of Agrobiotechnology, Centre for Research and Technology Hellas, Thessaloniki 57001, Greece; 3 Department of Genetics and Plant Breeding, Aristotle University of Thessaloniki AUTH, Thessaloniki 54006, Greece

## Abstract

**Background:**

Retrotransposons are key players in the evolution of eukaryotic genomes. Moreover, it is now known that some retrotransposon classes, like the abundant and plant-specific Sireviruses, have intriguingly distinctive host preferences. Yet, it is largely unknown if this bias is supported by different genome structures.

**Results:**

We performed sensitive comparative analysis of the genomes of a large set of Ty1/*copia *retrotransposons. We discovered that Sireviruses are unique among *Pseudoviridae *in that they constitute an ancient genus characterized by vastly divergent members, which however contain highly conserved motifs in key non-coding regions: multiple polypurine tract (PPT) copies cluster upstream of the 3' long terminal repeat (3'LTR), of which the terminal PPT tethers to a distinctive attachment site and is flanked by a precisely positioned inverted repeat. Their LTRs possess a novel type of repeated motif (RM) defined by its exceptionally high copy number, symmetry and core CGG-CCG signature. These RM boxes form CpG islands and lie a short distance upstream of a conserved promoter region thus hinting towards regulatory functions. Intriguingly, in the envelope-containing Sireviruses additional boxes cluster at the 5' vicinity of the envelope. The 5'LTR/internal domain junction and a polyC-rich integrase signal are also highly conserved domains of the Sirevirus genome.

**Conclusions:**

Our comparative analysis of retrotransposon genomes using advanced *in silico *methods highlighted the unique genome organization of Sireviruses. Their structure may dictate a life cycle with different regulation and transmission strategy compared to other *Pseudoviridae*, which may contribute towards their pattern of distribution within and across plants.

## Background

Retrotransposons and retroviruses (collectively referred as retroelements) can replicate their genomes via an RNA intermediate and insert the copies into new chromosomal locations of the host organism [1,2]. This 'copy and paste' process has the potential to greatly amplify their abundance, even over short evolutionary timescales, enabling them to become a major component of genomes [3-5]. Unlike retrotransposons, retroviruses have additional coding capacity in the form of an envelope (*ENV*) gene that allows them to enter the extracellular space and infect other individuals. Retrotransposons lack the *ENV *gene and cannot escape the cell, however they are free to reinfect their host genome. Long Terminal Repeat (LTR) retrotransposons form the most abundant transposable element type in plants, largely accounting for the vast differences in genome sizes [6]. Small genome plants like *Arabidopsis *(121 Mbp) and rice (389 Mbp) are sparsely populated by LTR retrotransposons, 5.6% [7] and 17% [8] respectively. In contrast, the LTR retrotransposon-derived fraction of medium/large genomes may reach up to 75% in maize (2.300 Mbp) [9,10] and 70% in barley (5439 Mbp) [11].

The two main superfamilies of LTR retrotransposons are the Ty1/*copia *(*Pseudoviridae*) and Ty3/*gypsy *(*Metaviridae*) [12], which differ in the order they package their genes in the coding domains. Both typically contain the *gag *gene and the *pol *gene region. *gag *encodes a capsid protein that forms the virus-like particle (VLP), which houses one or two RNA genomes and the enzymes for the cytoplasmic step of reverse transcription. *pol *encodes the enzymatic proteins required for the production of the DNA copy from the RNA template and the insertion of the new copy in the host genome: an aspartic protease (*AP*), integrase (*INT*), reverse transcriptase (*RT*) and RNaseH (*RH*) [13]. LTRs flank the retrotransposon genome and contain the *cis*-acting transcriptional regulators, the promoter and termination transcription points. The *cis*-acting boxes are often recognition sites of stress-related DNA binding factors (DBFs) and may be organized as arrays of two or three repeated motifs (RM) in tandem [14,15]. A 5' untranslated region (5'UTR) serves as the tether domain between the 5'LTR and *gag*, while the linker domain connects *pol *and the 3'LTR. At the junctions of the 5' and 3'LTR with the internal retrotransposon genome reside the primer binding site (PBS) and the polypurine tract (PPT), respectively, that prime cDNA synthesis during reverse transcription [16].

The International Committee on the Taxonomy of Viruses (ICTV) has classified Sireviruses into the *Pseudoviridae *family [17] together with the Pseudovirus and Hemivirus genera. It is the most recently described genus named after the SIRE1 element from soybean, and as they have colonized only plant species, they were originally named Agroviruses [13]. Sireviruses have putative retroviral properties, since many elements contain an *ENV*-like gene in their linker domain [18], which differentiates them from the other Ty1/*copia *genera (herein referred as 'classic' Ty1/*copia *elements, following the analysis of Havecker et al. 2005). Sireviruses have successfully proliferated within plant genomes, comprising a large proportion of the available Ty1/*copia *populations [19]. Examples of such elements are OPIE-2 and PREM-2 with more than 127,000 (fragmented) copies each in the maize genome, of which 3,530 and 4,093 are full length elements respectively [9], and Osr8 that is the second most abundant element in rice [8]. Phylogenetic analysis based on the *RT *domain revealed that Sireviruses are less diverse than classic Ty1/*copia *retrotransposons, indicating a possible recent evolutionary origin and colonization of their host genomes [20].

Sireviruses differ in the sequence and genomic organization compared to other elements. Whereas the vast majority of plant retrotransposons encode *gag *and *pol *as a single open reading frame (ORF), Sireviruses exhibit variation in respect to their organization. Besides the consensus, many elements have *pol *in two unusual +1 frameshifts relative to *gag*, of which the one suggests novel ways of translational recoding for *pol *expression, involving internal ribosomal entry or a bypass strategy [20]. For the bypass mechanism a highly conserved inverted repeat (IR) at the *gag*/*pol *boundary may provide a favorable secondary structure at the RNA level. Sireviruses also have a much larger *gag *gene compared to classic retrotransposons, which is characterized by a central RNA-binding CCHC zinc knuckle and a downstream predicted coiled-coil domain [13,18]. The larger capsid protein interacts with the plant Light Chain 8 protein family, which can bind to cargo like cellular proteins and aid their transportation, suggesting it may facilitate VLP assembly or movement towards the nucleus. So far, the evolutionary history of Sireviruses, their life cycle and why they are so successful in invading plant genomes remains unclear.

In this study, the in depth comparative analysis of *Pseudoviridae *genomes enabled the identification of several short but highly conserved sequence motifs in the otherwise diverse Sirevirus genome. The motifs are found in key regions of the non-coding genome, regions which are necessary for the activation, reverse transcription and integration of the element, or even its virulence capacity through the expression of the *ENV *gene. Interestingly, one of the motifs is semi-conserved in classic retrotransposons, implying a global fundamental role of its function. Our results suggest that Sireviruses are an ancient retrotransposon lineage that maintains core domains with absolute or high similarity. These domains appear to attribute novel features to the Sirevirus life cycle and may partially explain their distribution within and across plants.

## Results

### Sireviruses represent an ancient Ty1/*copia *genus

In order to establish a representative dataset for the comparative sequence analysis of *Pseudoviridae*, 21 Sireviruses were collected from various monocot and eudicot species including (Table S1 in Additional file 1): the five elements in the ICTV Sirevirus database (OPIE-2, PREM-2, SIRE1-1, ToRTL1, Endovir1-1); retrotransposons previously identified as Sireviruses, such as Osr7, Osr8, Osr9, Osr10 from rice [8], HOPIE from maize [21], Lotus2 from lotus, and one element each from sorghum, medicago and citrus [18,22]; plus Tnd-1 from tobacco [23], one *Vitis vinifera *retrotransposon and the Maximus lineage recently reported by Wicker and Keller (2007) that are re-classified as Sireviruses during this work. The classic Ty1/*copia *retrotransposon population consisted of 30 elements (25 from plants and five from other organisms), representing the other two genera that make up the *Pseudoviridae *family: 26 Pseudoviruses and four Hemiviruses (Table S1 in Additional file 1). Phylogenetic analysis based on the *RT *segment, which includes the 5^th ^to 7^th ^*RT *conserved domains [24] and continues through *RH *to the end of *pol*, separates the Sireviruses in a distinct clade with 100% bootstrap support (Figure S1 in Additional file 2). The relative shorter branch lengths of the Sirevirus clade compared to the other retrotransposons are in broad agreement with previous *RT *phylogenetic analyses [20], suggesting the recent colonization of their host genomes.

**Figure 1:**
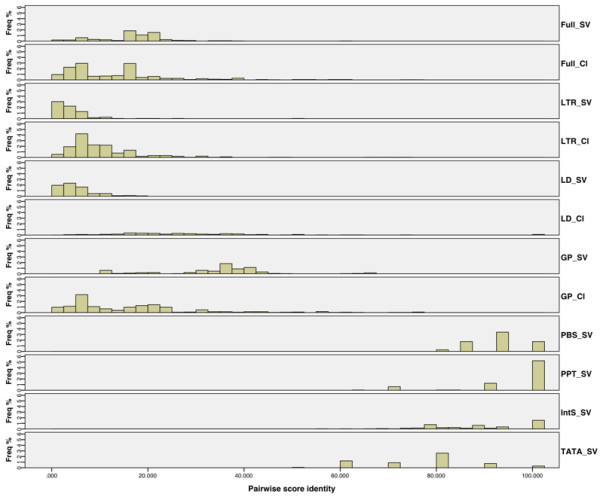
**Genome diversity of Ty1/*copia *retrotransposons**. Pairwise score identity of the various genomic regions of Sireviruses and classic elements and of the Sirevirus conserved motifs. Classic Ty1/*copia *elements with linker domain smaller than 25 bp are not included in the respective analysis. Most domains that are shared between the two retrotransposon datasets appear to be equally divergent (see also Table 1). SV, Sirevirus; Cl, Classic elements; Full, full length element; LD, linker domain; GP, *gag*-*pol*; PBS, conserved 5'LTR/internal domain junction; PPT, linker domain/3'LTR junction; IntS, integrase signal; TATA, TATA box-like motif.

Intriguingly, despite the closer evolutionary relationship than classic elements, the sequence organization of the Sirevirus genome appears to be highly divergent, as much as (or even more than) the genome of classic retrotransposons (Figure 1). In fact, excluding the coding domain, the non-coding regions are more diverse in Sireviruses (Table 1), which suggests that the mutation rates of genome change are similar or even higher in the Sirevirus genus compared to other *Pseudoviridae *retrotransposons. Consequently, the *RT*/*RH*-derived recent evolutionary origin of Sireviruses may be plasmatic and the genus may in reality represent an ancient lineage. Overall, the average pairwise identity of the full-length genome of Sireviruses and classic elements is equally low (17% and 13% respectively), whilst the Sirevirus linker domain and LTRs are extremely diverse (Table 1). Thus, the highly conserved motifs discussed herein are most likely not the result of general sequence similarity within the Sirevirus genome.

**Table 1:** Average pairwise score identity (%) of the various domains of Sirevirus (SV) and classic (Cl) elements, and also of the Sirevirus conserved motifs

Full length SV - Cl	16.6 - 13.2
LTR SV - Cl	3.9 - 10.5
Linker domain SV - Cl†	4.8 - 28.5
*gag*/*pol *SV - Cl	34.1 - 16.1
5'LTR/PBS junction SV	92.7
PPT/3'LTR junction SV	95.7
Integrase signal SV	88.2
TATA box SV	76.2

† Classic Ty1/*copia *with linker domain smaller than 25 bp are not included in the respective analysis.

### Multiple identical PPTs is a unique feature of the Sirevirus linker domain

**Figure 2:**
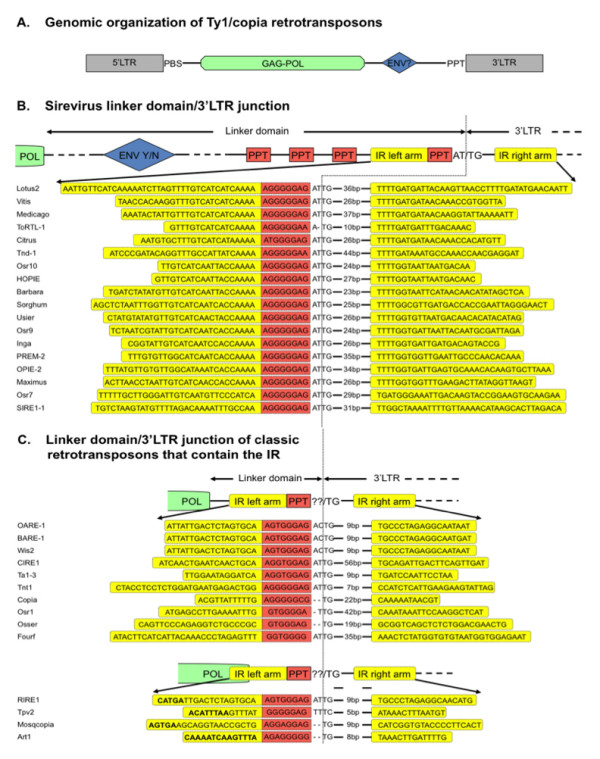
**The organization of the linker domain/3'LTR junction of Ty1/*copia *retrotransposons**. (A) Genome structure of Ty1/*copia *retrotransposons, of which Sireviruses may contain an envelope-like gene. (B, C) Organization of the Sirevirus and classic elements linker domain/3'LTR junction. The IR arms (yellow) surround the loop sequence, of which the terminal PPT octamer (red) occupies the outmost 5' side. Classic elements with nucleotides in bold indicate the region, where the left arm is located at the 3' end of the *pol *gene region, due to the very short linker domain.

Unsupervised discovery of sequence motifs with the use of the TEIRESIAS pattern discovery algorithm [25] primarily identified the downstream 3' end of the internal domain as the most conserved area of the Sirevirus genome, which corresponds to the region where the PPT is located. Post-discovery analysis, however, differentiated the area into two discrete sub-regions, the PPT and an IR, that have different characteristics. The first motif is the 8 bp 5'-AGGGGGAG-3' sequence, which is found precisely 10 bp upstream of the 3'LTR of all elements. TEIRESIAS detected many identical PPT copies in the linker of every Sirevirus, irrespective of the length of the domain or the presence of the *ENV*-gene (Figure 2b and Table 2). On average, four PPTs are found in each linker, preferentially clustered near the 3'LTR junction (83% within 0.5 kb from the 3'LTR). The biased multiple PPT topology is exemplified in the *ENV*-containing Sireviruses, where the vast majority of upstream PPTs (56/63, 89%) reside after the *ENV*-gene (Table 2). The presence of the PPTs in the linker domain is not coincidental, as the octamer is rarely found in the rest of the Sirevirus genome (0.3/element). We searched for variants of the 8 bp motif with no significant results, which suggests that sequence integrity is essential and under selection. ToRTL1 and Tnd-1 are the only two Sireviruses that show variation in their PPT nucleotide composition compared to the consensus octamer. Nevertheless, both elements have the terminal G replaced by an A in all their PPTs, which also points towards strong selective pressure instead of random substitutions within the motif.

**Table 2:** Analysis of the Sirevirus genome size, the position and number of PPTs

Element	Full/Linker/LTR length (kb) †	PPTs before *ENV*	PPTs after *ENV*	PPTs in linker domain
Lotus2	12.12/1.68/1.22	---	4	4
Osr10	11.97/2.04/1.56	1	3	4
Osr9	11.63/1.68/1.51	---	4	4
Sorghum	12.10/1.79/1.22	1	3	4
Medicago	11.93/1.14/1.27	---	4	4
Citrus	9.98/1.28/1.16	---	5	5
Hopie	11.87/1.37/1.68	---	3	3
ToRTL1	9.68/0.88/0.80	---	4	4
SIRE1-1	9.29/0.52/1.00	---	5	5
Endovir1-1	9.08/1.55/0.54	---	3	3
Vitis	9.67/1.10/1.24	1	3	4
Barbara	9.90/2.76/1.66	1	3	4
Maximus	14.42/3.96/1.41	---	4	4
Inga	12.02/1.61/1.43	3	2	5
Usier	11.77/1.84/1.57	---	3	3
ATCOPIA43	9.07/1.59/0.56	---	3	3
Osr7	8.92/1.31/1.62	5		5
Osr8	9.21/1.10/1.21	3		3
Tnd-1**††**	8.45/0.73/1.59	4		4
OPIE-2	8.98/0.79/1.29	4		4
PREM-2	9.43/0.82/1.30	5		5
**Avg of Sireviruses**	10.55/1.50/1.28			4/element
**Avg of classic *copia***	5.60/0.078/0.56	All elements have 1 PPT in their linker		

**† **For elements that contain the *ENV*-gene, the linker domain length was calculated by combining the segments before and after the *ENV*-ORF. **†† **The 5'LTR of Tnd-1 is incomplete and therefore the full length is calculated based on the 3'LTR.

**Figure 3:**
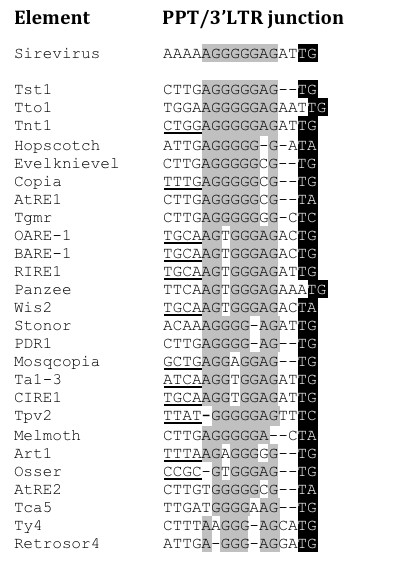
**The diversity of the PPT/3'LTR junction of classic Ty1/*copia *retrotransposons**. Alignment of the conserved Sirevirus PPT/3'LTR junction to the respective region of classic Ty1/*copia *retrotransposons. Underlined bases represent the 3' terminus of the IR left arm, and nucleotides shaded in black indicate the beginning of the 3'LTR. The location of the IR left arm is universally conserved in all *Pseudoviridae*.

Conversely to Sireviruses, TEIRESIAS did not discover any conserved motifs in the linker domain and 3'LTR of classic retrotransposons. All elements have a single terminal PPT with a wide range of sequence variation, which does however often resemble the Sirevirus PPT (Figure 2c and Figure 3). Three elements share the Sirevirus octamer, while 17 differ only in one nucleotide. The most common substitutions are the conversions of the G of the 3^rd ^position to a T and the A of the 7^th ^position to a C.

### A highly conserved attachment site defines the Sirevirus terminal PPT

The attachment site consists of a short sequence, usually a di- or trinucleotide, between the PPT and the 3'LTR, and the first few bases, especially the universal dinucleotide TG, of the start of the 3'LTR. It is recognized and processed by *INT *during the successful insertion of the new copy into the host genome [26]. TEIRESIAS led us to observe that in the Sirevirus 3'LTR junction the conserved motif includes not only the PPT octamer, but also the following four bases. In 20 elements the terminal PPT is followed by the intervening dinucleotide AT, whilst the universal TG indicates the beginning of the 3'LTR, thus generating the Sirevirus 5'-AT/TG-3' attachment site (Figure 2b). The overall nucleotide identity of the combined twelvemer is 95.7%. Interestingly, the sequence following the upstream PPTs is highly variable, as only four octamers (out of 63) employ an AT dinucleotide, of which none is linked with the TG motif that would create a putative functional attachment site. In contrast, classic retrotransposons have a highly variable attachment site, with some elements lacking intervening bases between the terminal PPT and the 3'LTR (Figure 2c and Figure 3).

### A precisely positioned inverted repeat surrounds the PPT/3'LTR junction of Sireviruses and some classic retrotransposons

As previously mentioned, TEIRESIAS included bases immediately upstream of the terminal PPT within the most conserved motif of the Sirevirus genome. Subsequent analysis revealed that this upstream region constitutes the 3' section of a sequence (left IR arm), which is found inverted within the 3'LTR (right IR arm). The IR is present in all but three elements (Osr8, Endovir1-1 and ATCopia43) and shares high nucleotide identity even between Sireviruses that colonize evolutionary distant monocot and eudicot plant species (Figure 2b). The main characteristic of the motif is the precise position of the left arm, which always borders the PPT octamer. The intervening 'loop' sequence is short in length and consists in a 5' to 3' direction of the terminal PPT, the 5'-AT/TG-3' attachment site and the beginning of the 3'LTR, which is diverse with large parts often deleted. The right arm starts on average 28 bp inside the 3'LTR. Each element contains highly similar arms (on average 89% identity, 28 bp length), however the sequence conservation is higher in the region surrounding the loop and decreases rapidly towards the periphery of the motif (Figure S2 in Additional file 2). The upstream PPTs lack an IR structure, which suggests that any putative function of the motif is available only through the natural PPT/3'LTR junction.

We discovered that the IR also occurs in almost half classic retrotransposons (Figure 2c), indicating that this structure may serve a fundamental role in the life cycle of the *Pseudoviridae*. In all cases the left arm extends downstream till the beginning of the predicted PPT, which occupies the 5' border of the loop sequence (Figure 3). This precise arrangement offers great support to the proposed PPT for classic retrotransposons. The IR length within each element is considerably smaller compared to Sireviruses (on average 88.6% identity, 19 bp length) (Figure S3 in Additional file 2), whilst the right arm starts on average 18 bp inside the 3'LTR. Characteristically, in elements with very short (RIRE1, Tpv2, Mosqcopia) or absent (Art1) linker the left arm resides partially or completely in the coding domain (nucleotides in bold, Figure 2c).

### A novel type of repeated motif (RM) resides in the Sirevirus LTRs and the region upstream of the *ENV*-gene

**Figure 4:**
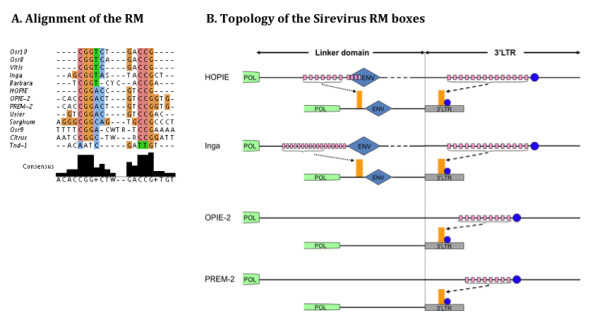
**Sequence composition and distribution of the novel Sirevirus RMs**. (A) Alignment of the Sirevirus palindromic RM boxes (refer to Table 3 for the intervening bases of each RM), (B) and their organization (pink boxes) in four elements at the 5' side of the *ENV *gene (only HOPIE and Inga), and upstream of the TATA box (blue circle) in the 3'LTR. All or a section of the RM clusters define the borders of CpG islands (orange bar) (all elements are shown in Figure S4 in Additional file 2)

We identified in Sireviruses a highly repeated motif with distinctive characteristics in two key areas of the genome of LTR retrotransposons [2], which may indicate its involvement in the regulation of Sirevirus activity. The RM is found in 13 elements and is a Sirevirus specific feature, as it is absent from the genome of classic retrotransposons (Table 3). Besides being abundant in the LTRs, the existence of the *ENV*-gene in nine elements is intriguingly concomitant to the presence of additional RM copies in the linker domain (Table 3, Figure 4b and Figure S4 in Additional file 2). In fact, the distribution of these copies contradicts the multiple PPT topology as the vast majority (77/79, 97%) are preferentially located upstream of the *ENV*-gene (Table 2 and 3). It appears, therefore, that for these copies the *ENV*-gene is the determining factor. Moreover, in Osr10 and HOPIE there are extra copies at the beginning of the *ENV*-gene, near the junction with the linker domain. In contrast, the four Sireviruses without the *ENV*-gene are simultaneously devoid of linker-based copies. The distribution of the RMs in the Sirevirus 3'LTR is also non-random. Most of the copies (117/127, 92%) cluster within the upper half of each LTR, as they start 200-400 bp after the PPT/3'LTR junction and extend to the following 300-500 bp. As much as 32 units can occur in one element, while all Sireviruses contain at least eight copies of their RM. The overall nucleotide conservation within most elements is remarkable, commonly displaying more than 85% identity (Table 3).

**Table 3:** Sequence, copy number and nucleotide identity of the 13 novel RMs

Element	Total copy number	*ENV*	Linker		3'LTR	Consensus sequence††	Nucleotide identity %
			bef. *ENV*	aft. *ENV*			
Osr10**†**	22 (+1)	Y	12	0	10	CGGTCTGACCG	92.7
Osr9	20	Y	7	0	13	TTTTCGGACWTRTCCGAAAA	88.1
Sorghum	9	Y	4	2	3	AGGGCGGCAGTGCCGCCCT	80.1
Citrus	12	Y	8	0	4	AATCCGGCTWRCCGGATT	66.5
HOPIE**†**	21 (+4)	Y	7	0	14	CGGACCGTCCG	86.8
Vitis	12	Y	2	0	10	CGGTCGACCG	87.4
Barbara	26	Y	13	0	13	TCGGTCYCACCGA	85.2
Inga	32	Y	18	0	14	AGCGGTASTACCGCT	87.4
Usier	18	Y	6	0	12	GTCGGACGTCCGAC	76.1
OPIE-2	9	N	0		9	CACCGGACTGTCCGGTG	100
PREM-2	8	N	0		8	CACCGGACTGTCCGGTG	95.5
Osr8	9	N	0		9	CGGTCAGACCG	85.4
Tnd-1	8	N	0		8	ACAATCGATTGT	100

**† **The parenthesis includes the additional copies found at the beginning of the *ENV*-gene. **†† **The symmetrical subunits of each RM are underlined, revealing the length and composition of the intervening bases.

The sequence of all RMs is palindromic with dyad symmetry structure (Table 3), which often characterizes retrotransposon *cis*-acting transcriptional activators [14]. Besides Tnd-1, the RMs of the other Sireviruses contain a CGG-CCG signature that forms their core structure and generates the palindromy (Figure 4a). The highly conserved signature comprises the absolute base of homology, irrespective of the presence of intervening nucleotides between the two symmetrical subunits, or the additional sequence that participates in the symmetry. As an example, Osr10, Osr8, Vitis and Inga share nearly identical RMs, although they have very low sequence homology and reside in evolutionary distant monocot and eudicot species. When we compared the RMs against known plant *cis*-acting regulatory sequences using the PLACE database [27], we mainly recovered stress-related transcription factor recognition sites (Table S2 in Additional file 3). Possibly then, stress-related stimuli may regulate the Sirevirus activation, although experimental confirmation like RM:promoter:GUS fusion analysis is required to correlate the RMs to any regulatory function.

### Clusters of RMs in the LTRs and upstream of the *ENV*-gene define the borders of predicted CpG islands

CpG islands are known to be strongly associated with genes, as they typically occur within or close to their 5' terminus near the transcription start site and have been found both in vertebrates [28] and plants [29]. Their unmethylated status has been involved with the regulation of gene expression [30]. The Sirevirus genome seems to contain CpG islands, which are preferentially found in the LTRs and the region upstream of or within the 5' domain of the *ENV*-gene. Of the 21 elements, Tnd-1, Sorghum and ToRTL1 lack CpG islands, while only twice (in *pol *of Osr9 and Osr8) does a predicted island occur in a region other than the LTRs or the *ENV*. Among the RM-containing Sireviruses, the RM clusters of HOPIE, OPIE-2, PREM-2, Vitis, Barbara, Inga and Usier are the only CpG islands in their genome, whilst one or two additional islands are present in Osr10, Osr9, Citrus and Osr8 (Figure 4b and Figure S4 in Additional file 2). Presumably, the conserved and high copy number CGG-CCG signature is the key motif of the islands. In most occasions the first and last units of the RM cluster precisely define the borders of the CpG islands, which by itself supports the hypothesis that they may have a role in the Sirevirus regulatory network. Excluding one very long island in the *ENV *region of Usier that continues far beyond the last RM, the average 400 bp Sirevirus CpG island starts and finishes approximately 45 bp upstream and downstream of a RM unit, respectively. In contrast to the positional stability of the Sirevirus CpG islands that imply their functionality, most classic elements do not contain a similar pattern (not shown). Only seven have LTR-derived islands, whilst nine lack such a domain and 14 have irregular conformations where CpG islands are present in varied non-LTR locations.

### A conserved TATA box lies shortly after the CpG island in the diverse Sirevirus LTR

**Figure 5:**
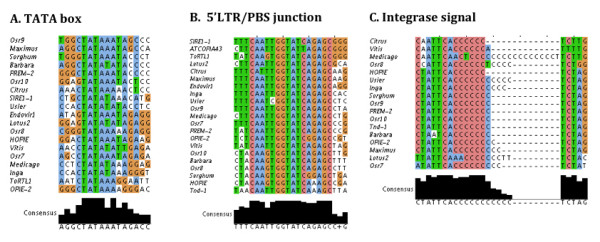
**Alignment of the Sirevirus conserved TATA box region (A), 5'LTR/internal domain junction (B), and polyC-rich integrase signal (C)**.

Another feature that differentiates Sireviruses from classic retrotransposon genomes is the presence of a conserved TATA box motif in the Sirevirus LTRs. The promoter element 5'-cTATAA/TAT/AAg-3', where lower case letters represent less critical bases [31], resides in all but two elements (Tnd-1 and ATCOPIA43) (Figure 5a) and is approximately located in the middle of each LTR (average nucleotide identity 76%). Intriguingly, in the RM-containing Sireviruses the TATA box occurs on average 37 bp after the CpG island, which suggests that the motif corresponds to an authentic promoter and the proximal RM units may regulate its activation (Figure 4b and Figure S4 in Additional file 2).

### Conservation of the Sirevirus 5'LTR/internal domain junction

The second most popular sequence motif of the Sirevirus genome according to TEIRESIAS is the 5'LTR/internal domain junction that accommodates the PBS. The highly conserved upstream junction is divided in three subdomains that in the 5' to 3' direction consists of the universal CA end of the 5'LTR, the intervening sequence and the PBS (Figure 5b). The Sirevirus PBS is only 9 bp (5'-TATCAGAGC-3'), complements the 3' end of the ^met ^tRNA, which is therefore the first primer during reverse transcription. The sharp decrease in sequence conservation after the ninemer implies that Sireviruses paradoxically use only a short segment of the tRNA molecule to prime cDNA synthesis. The intervening sequence is unusually long (5'-ATTGG-3') and in six elements a G replaces the T at the second position. Consequently, the Sirevirus upstream attachment site is the heptamer 5'-CA/ATTGG-3', whilst over the whole length of the 16 bp region the genus maintains 92.7% identity. In contrast, classic elements contain a more variable 5'LTR/PBS junction, although and due to the common ^met ^tRNA-derived PBS in many LTR retrotransposons [32], TEIRESIAS discovered a semi-conserved sequence motif in some elements (data not shown).

### A polyC-rich integrase singal is located at the 3' end of the Sirevirus LTRs

The successful integration of a new retrotransposon copy in the host genome is highly dependent on the interactions between *INT *and the attachment sites of the element [26]. However, research on retroviruses has shown that binding specificity of *INT *to the viral DNA is achieved through additional interactions that extend at least 25 bp inwards at the 3' end of the LTR in a domain that is called the 'integrase signal' [12,33]. TEIRESIAS identified a conserved motif in this location in 17 Sireviruses. The putative Sirevirus integrase signal is approximately 19 bp long (nucleotide identity 88.2%) and contains a distinctive C-rich region in the central domain that varies in size (Figure 5c). The average distance between the signal and the LTR end is 21 bp. To our knowledge this is the first time such a motif, which also characterizes a whole genus, is reported in the genome of LTR retrotransposons.

### Sireviruses are the 'obese' members of the *Pseudoviridae *family

A comparative analysis of the size between Sireviruses and classic elements revealed that Sireviruses share an exceptionally large genome that clearly differentiates them from other Ty1/*copia *retrotransposons (Table 2). So far few publications have commented on the length of single Sirevirus elements, the most recent reporting on the large genome of the Maximus lineage [34], ignoring however that Maximus belongs to the Sirevirus genus. The average Sirevirus length (10.55 kb) is twice as long as that of classic Ty1/*copia *elements (5.6 kb). The difference derives from the large LTRs (1.28 kb compared to 0.56 kb), linker domain (1.5 kb to 78 bp), expanded *gag *(660 to 330 amino acids) and *ENV *(1.8 kb when present). The sum of the extended regions equals to 5.5 kb and hence matches the size difference.

## Discussion

**Figure 6:**
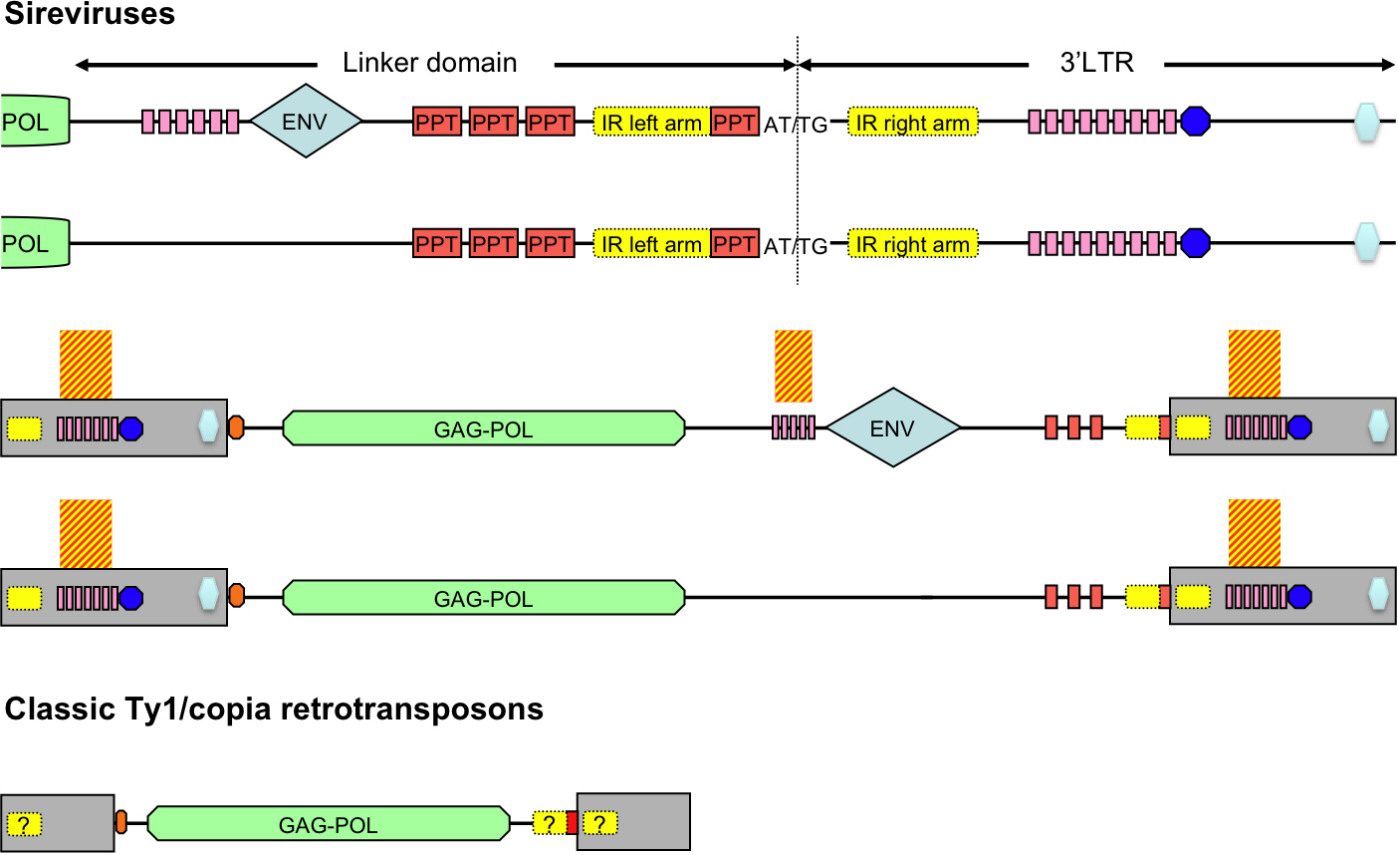
**The organization of the highly conserved microdomains in the Sirevirus genome**. The coloring follows the pattern of previous figures. The conserved 16 mer of the 5'LTR/PBS junction is shown in orange, whilst the integrase signal at the 3' terminus of the LTRs in light blue. The size difference of Sireviruses and classic retrotransposons is approximately drawn to scale (see also Table 2).

The novel *in silico *analysis of the Sirevirus genome and the comparison to a group of representative classic elements provided what we believe are major insights into the structure of LTR retrotransposons. Our data show that Sireviruses form an ancient lineage, at least as old as those of other classic Ty1/*copia *elements. The main characteristic of their genome is the high sequence divergence of their large non-coding domains, which are however full of highly conserved microdomains in key regions (Figure 6) that indicate participation in every step of the retrotransposon life cycle, from activation to reverse transcription to integration in the new locus. The position and organization of the motifs do not seem coincidental: i) the novel RMs define CpG islands at the *ENV *5' terminus and in the LTRs adjacent to the TATA box, ii) the junctions contain highly conserved PBS, PPT and attachment sites, whilst an IR accurately surrounds the terminal PPT, iii) multiple identical PPTs cluster next to the 3'LTR and iv) the integrase signal occurs at the predicted domain at the 3' end of the LTRs. These features may be among the underlying factors that contribute towards the Sirevirus distribution in plants.

### The high conservation of the Sirevirus LTR junctions indicates strict sequence requirements towards reverse transcription and integration

The PPT and the pairing of the tRNA molecule to the PBS are used as primers during reverse transcription to initiate plus and minus strand cDNA synthesis, respectively. Both steps are followed by template switching events (or jumps) that transfer the partial DNA fragments to the other side of the template, so as to resume and complete cDNA synthesis [16]. During the jumps the intervening nucleotides inbetween the 5'LTR-PBS and PPT-3'LTR junctions are ordinarily added to the outer sides of the linear cDNA intermediate, hence generating the attachment sites. In the first step of integration in the new genomic location, namely the 3' end processing, *INT *recognizes and cleaves the intervening nucleotides from the 3' ends of the linear cDNA, thereby preparing the new copy for nucleophilic attack [26].

According to the TEIRESIAS algorithm the two most conserved motifs of the Sirevirus genome are the PPT/3'LTR and 5'LTR/PBS junctions. The extreme sequence conservation of the two domains, which appear to be very important for reverse transcription and integration [2], reflects the strict sequence requirements of the Sirevirus replication strategy. Also, the presence of the integrase signal is unique among LTR retrotransposons and suggests the tight cooperation of *INT *with the Sirevirus LTRs. In contrast, the respective regions of classic elements show extensive sequence variation, although a large proportion maintains a common PPT structure that relates to the Sirevirus PPT octamer. We propose that the primer for plus strand cDNA synthesis in the majority of *Pseudoviridae *consists of eight bases, which may tolerate some variability in classic elements, but is strictly conserved in Sireviruses.

### Which is the role of the multiple PPTs?

The presence of multiple PPTs within retroelements is not unprecedented, however their role in plus strand cDNA synthesis is unclear. One extra PPT is present in the linkers of the *ENV*-containing Ty3-*gypsy *elements Athila4 and Calypso [35], whilst one functional PPT has been reported in the coding domains of Ty1 and HIV1 [36]. PPT-primed cDNA synthesis is a rapid event and when extra PPTs occur in the coding domain, synthesis initiates simultaneously with the natural PPT [37]. Yet, the upstream-primed cDNA must synthesize several kilobases of sequence and as a result it is outcompeted by the natural PPT-primed cDNA due to kinetic competition [36]. In Sireviruses kinetic competition is unlikely to prevent strand transfer, since the distance between the PPTs is small. The critical parameter might be the availability of the tRNA primer at the 3' end of the minus DNA strand that is utilized during the second strand transfer to provide complementarity to the PBS sequence at the 5' end of the minus DNA strand. Rapid *RH *degradation of the tRNA after it is copied in the natural PPT-primed cDNA may prohibit strand transfer from internally initiated cDNAs [36].

The upstream PPTs also lack the correct attachment site and the IR motif, which further obscures their functionality. The transposition ability of the retroelement is dependant on the attachment site [26]. If the PPT is not followed by the correct signal for *INT *processing, the cDNA intermediate will be unable to insert in the new genomic location. On the other hand, the strict nucleotide integrity of the upstream PPTs, their high copy number and preferential clustering next to the 3'LTR point towards selection. They may facilitate genetic recombination between the two RNA genomes in the VLP [38], or offer a relative replication advantage to the genus [39]. In any way, based on the fact that retrotransposons with *ENV *genes contain near their 3'LTR repeats that show similarity to PPTs, Havecker et al. (2004) proposed their involvement in reverse transcription.

### The IR can act as facilitator for the correct PPT primer formation

Research in Ty1 has shown that the PPT sequence by itself is not sufficient to promote correct *RH *processing. Additional recognition signals in the form of secondary structures near the PPT domain may be needed to affect the overall conformation of the region and attract the *RH *for PPT formation and subsequent DNA elongation [37]. A highly conserved IR that forms a stem loop secondary structure, of which the 8 bp PPT retains its outmost left side, seems as the most suitable candidate for such a recognition signal. Presumably, this configuration preferentially 'exposes' the terminal PPT and renders it accessible to *RH *for precise cleavage, which will automatically generate the highly conserved attachment site. Many classic Ty1/*copia *elements also contain the IR, which suggests that this motif may have a universal role in retrotransposon life cycle.

### Are the RM boxes involved in the regulation of Sirevirus activity?

Sireviruses share highly similar versions of a novel RM, which in conjunction to the exceptional high copy number and flanking sequence divergence, make the conservation remarkable. Symmetry is a frequent feature of regulatory sequences, including box I of Tnt1 [14], an activator in the 5'UTR of Copia element [40] and the Pal elements in the β-glucokinase promoter of neuroendocrine pancreatic, gut and brain cells in humans and mice [41]. The position of the boxes in the LTRs and upstream of the *ENV*-gene does not seem random and they also form CpG islands that are by default found at the 5' side of genes regulating their expression [29]. Interestingly, Sireviruses lacking the boxes may have similar repeated structures, as large sections of their LTRs and upstream of the *ENV *are particularly CG-rich and generate CpG islands (data not shown). Despite these unique characteristics, we could not identify a common binding site targeted by a single DBF. It appears that minor variation, notably the presence of one intervening base or a substitution in the symmetrical motif, may confer different binding properties that may enable interactions with different nuclear factors. Future promoter analysis experiments could elucidate the role, if any, of the RMs in the Sirevirus activation network.

### Clues into the functionality of the envelope gene

The function of the *ENV*-gene in plant retroelements has not been experimentally proven and due to the plant cell wall its role remains highly controversial [42]. So far it has been known that *ENV*-containing plant retroelements express their *ENV *either through a spliced *ENV*-like mRNA, for example splice acceptor sites have been predicted for the Ty3-*gypsy *BAGY2 and Athila elements [35,43], or through stop codon suppression in SIRE1 [44]. However, SIRE1 is an atypical Sirevirus, as *pol *and *ENV *are separated only by a stop codon. The sequence of the Sirevirus *ENV*-gene is highly variable [18]. Peterson-Burch and Voytas (2002) hypothesized that the Sirevirus *ENV *may be expressed from an internal promoter, although they were not able to identify promoters or transcription factors-binding sites upstream of the *ENV*. Our work indicates that specific motifs, which are also the epicentre of CpG islands, may have a regulatory role in the 5' vicinity of *ENV*, which offers support to the internal promoter hypothesis for *ENV *expression, although we were not able to conclusively identify one. Intriguingly, CpG islands are predicted in the same domain for six *ENV*-containing Sireviruses that lack the RM boxes, even in the *pol*/*ENV *junction of SIRE1-1. Collectively, we believe our observations to be the first set of evidence concerning the Sirevirus *ENV *expression and their potential capacity to become virulent during their life cycle.

### Are Sireviruses capable of horizontal transmission?

Sireviruses are abundant and widely dispersed in plants [19]. Moreover, it is the only *Pseudoviridae *genus to contain an *ENV *gene, which is hypothesized as being funtional. Has horizontal transmission, which is a sporadic incident, aided their proliferation? Possibly as plant viruses do, Sireviruses accumulate in the cytoplasm within their envelope and wait for a feeding invertebrate to ingest and transfer them to another plant [35]. If horizontal transmission has occurred at high rates, then one would expect to frequently encounter elements with high nucleotide similarity in diverse plant phyla, which does not seem to be the case. However, this observation does not rule out the possibility that horizontal transfer can rarely happen, but when it does occur, Sireviruses quickly colonize the new host assisted by their unique genomic organization. Alternatively, the Sirevirus envelope or *gag *capsid may act as a retrovirus movement protein and enable VLP transport outside the cell to infect different tissues of the same host, including meristem or germ cells, thereby enhancing the vertical transmission rate [45,46].

### The distinctive relationship of the maize Sireviruses OPIE-2 and PREM-2

The conservation of the motifs is exemplified in the closely related OPIE-2 and PREM-2 maize elements. The alignment of their LTRs provides striking results, as the microdomains are the only highly similar segments amidst diverse sequences (Figure S5 in Additional file 2), often even higher than regions of *pol *and particularly *gag*. These contradict the sequence differentiation that has been reported for the Tnt1 superfamily and to the best of our knowledge this is the first time such a pattern of sequence conservation is shown. The Tnt1 superfamily consists of retrotransposons with surprisingly homologous genomes that colonize species of the Solanaceae family [47,48]. The only region that exemplifies high genomic variability is the LTR domain that harbors the *cis*-acting motifs [49,50]. The sequence divergence and evolution of the motifs has equipped the Tnt1 superfamily with high adaptive regulatory capacity that allowed subgroups to evolve differently and colonize new Solanaceae genomes [51,52]. It appears, therefore, that the OPIE-2/PREM-2 conservation pattern contradicts those of the Tnt1 superfamily and implies that the two elements may respond to the same stimuli and have the same transmission strategy.

## Conclusions

Aided by the novel computational analysis which was orchestrated by the TEIRESIAS pattern discovery algorithm, we identified a plethora of highly conserved motifs in specific non-coding regions of the Sirevirus genome (Figure 6) and we hypothesized that these may be among the factors that underlie the extensive propagation of the genus in plant species. The motifs may be involved in the activation of the element and the *ENV*-gene, but also influence the folding of the RNA and DNA strands, suggesting their interaction with *gag *and the *pol *enzymes during VLP assembly, reverse transcription and integration. Based on the extreme sequence divergence outside the coding domain, we proposed that Sireviruses form an ancient lineage, which challenges previous research that argued in favour of the young evolutionary origin of the genus. Amidst their diverse non-coding genome, strict functional pressures apparently act on the motifs to maintain their sequence integrity. The degree of conservation is astonishing, as the Sirevirus elements of our dataset reside in plant species that diverged 140-150 Mya (monocots/dicots) [53].

As vast sequence data become available from whole-genome sequencing projects, the findings of this work should enable the detailed analysis of how Sireviruses interact with their plant hosts and shape their genomes, towards which the fact that Sireviruses inhabit only plants may have important implications. While our knowledge on the evolutionary depth, distribution and genomic organization of Sireviruses will expand, more informed functional analysis of the herein introduced motifs will elucidate how and why the Sirevirus life cycle differs from other retrotransposons. In light of such knowledge, in the modern era of large-scale transcriptome analysis, and in possession of new evidence for the major influence of retrotransposon acticity on the cell transcriptional output (alternative promoters, noncoding RNAs, bidirectional transcription) [54,55], we should be able to move closer to elucidating the role of Sireviruses in the epigenetic regulation of plant genes [56]. Finally, based on recent models that propose transposable elements as being major suppliers of *cis*-regulatory elements to host genomes [57], Sireviruses with their novel type of RM may have been involved in the evolution of plant gene regulatory networks.

## Methods

### Ty1/*copia *datasets

The Sirevirus and classic retrotransposon datasets were constructed by retrieving sequences from various sources. The information for each element, including the source, host species and *Pseudoviridae *genus is provided (Table S1 in Additional file 1). The *Pseudoviridae *classification according to the ICTV database can be found at http://www.ncbi.nlm.nih.gov/ICTVdb/ICTVdB/00.097.htm. The raw sequence data (where all the conserved motifs are highlighted) of our Sirevirus database is available at http://bat.ina.certh.gr/downloads/Sirevirus_sequence_data.doc.

### Sequence analysis tools

Multiple alignments were carried out using ClustalW at default parameters [58] and the Jalview multiple alignment editor [59] from the EBI website http://www.ebi.ac.uk/. MEGA4 [60] was used to construct the phylogenetic tree using the neighbor-joining distance method [61]. Bootstrap test of phylogeny was calculated based on 1000 replicate trees and the evolutionary distances were computed using the Poisson correction model [62]. The *RT*-*RH *peptide alignment used for the analysis is provided (Additional file 4). Identification of the RM boxes and the IR were aided by the Tandem Repeat Finder [63] and Inverted Repeat Finder algorithms [64], respectively, available at the Laboratory for Biocomputing and Informatics http://tandem.bu.edu/tools.html. The RMs were compared against known plant *cis*-acting regulatory sequences in the PLACE database [27,65], in order to identify target sites of DBFs. Finally, CpG islands were predicted using the CpGplot package [66] from the EBI website.

### TEIRESIAS pattern discovery algorithm

Discovery of sequence motifs was performed with the use of the TEIRESIAS algorithm [25]. Importantly, TEIRESIAS is guaranteed to report all maximal patterns (i.e. patterns that cannot be made more specific and still keep on appearing at the exact same positions within the input sequences, or else non-redundant patterns) meeting the structural restrictions set by the user through parameters. In other words, no heuristics are employed in the discovery process. The complete set of parameters of TEIRESIAS used in this analysis is as follows: nucleotides in the pattern (-l), number of overlapping characters in the convolved pattern (-c), maximum length of an elementary pattern (-w), minimum number of appearances of the pattern (-k), flag for the support k to be the minimum number of sequences in which a pattern should appear (compared to the minimum number of instances of the pattern) (-v). Several sets of values for l, w, and k were used to obtain a comprehensive picture of the conservation of motifs in the input and this information was used critically and in context to produce the final observations.

## Abbreviations

PPT: polypurine tract; LTR: long terminal repeat; ENV: envelope; VLP: virus-like particle; AP: aspartic protease; INT: integrase; RT: reverse transcriptase; RH: RNaseH; DBF: DNA binding factor; RM: repeated motif; UTR: untranslated region; PBS: primer binding site; ICTV: International Committee on the Taxonomy of Viruses; ORF: open reading frame; IR: inverted repeat.

## Authors' contributions

AB conceived and designed the study, carried out the data collection, analysis, and interpretation, and drafted the manuscript. ND carried out pattern discovery with the TEIRESIAS algorithm, assisted in data interpretation and critically revised the manuscript. AT assisted in data interpretation and critically revised the manuscript. SP conceived and designed the study, and critically revised the manuscript. All authors read and approved the final manuscript.

## Supplementary Material

Additional file 1 **The *Pseudoviridae *dataset used in this analysis. **This file contains supplementary Table S1 showing the source, accession number, host species and *Pseudoviridae *genus for each retrotransposon. Click here for file

Additional file 2 **Phylogenetic and structural domain analysis of the Sirevirus and classic Ty1/*copia *retrotransposons. **This file contains supplementary Figure S1 showing the phylogenetic analysis of the Ty1/*copia *retrotransposons based on the *RT*/*RH *domains, supplementary Figures S2 and S3 with alignments of the IR arms of each Sirevirus and classic retrotransposon respectively, supplementary Figure S4 showing the distribution of the novel Sirevirus RM boxes, and supplementary Figure S5 with the 5'LTR alignment of the OPIE-2 and PREM-2 Sireviruses. Click here for file

Additional file 3 **The stress-related DNA binding factors that target core sites within the RMs. **This file contains supplementary Table S2 with information related to the binding site and its orientation within each RM and the stress nature of the DBFs. Click here for file

Additional file 4 List of the *RT*/*RH *peptide sequences that were used for the construction of the Ty1/*copia *phylogenetic tree. Click here for file
