# Supplementary material for: Highly conserved motifs in non-coding regions of Sirevirus retrotransposons: the key for their pattern of distribution within and across plants?
Source: BMC Genomics. 2010 Feb 4;11:89. doi: 10.1186/1471-2164-11-89 (PMC2829016; doi:10.1186/1471-2164-11-89)
Supplement: Additional file 1 — The Pseudoviridae dataset used in this analysis. This file contains supplementary Table S1 showing the source, accession number, host species and Pseudoviridae genus for each retrotransposon. [file 1471-2164-11-89-S1.PDF]

Table S1. The *Pseudoviridae* dataset used in this analysis

Element	Source/Accession number	Species	*Pseudoviridae* genus
Lotus2	retrieved manually from BAC AP009625	*Lotus japonicus*	Sirevirus
Osr10	http://www.public.iastate.edu/~voytas/MSsupplementary/Gag_LC8/	*Oryza sativa*	>>
Osr9	>>	*Oryza sativa*	>>
Sorghum	retrieved manually from BAC AF503433	*Sorghum bicolor*	>>
Medicago	retrieved manually from BAC AC130810	*Medicago truncaluta*	>>
Citrus	retrieved manually from BAC AF506028	*Citrus*	>>
Hopie	retrieved manually from BAC AC116033	*Zea mays*	>>
ToRTL1	U68072	*Lycopersicon esculentum*	>>
SIRE1-1	AF053008	*Glycine max*	>>
Endovir1-1	AY016208	*Arabidopsis thaliana*	>>
Vitis	retrieved manually from BAC AM424501	*Vitis vinifera*	>>
Barbara	http://wheat.pw.usda.gov/ITMI/Repeats/ TREP_3456	*Triticeae*	>>
Maximus	http://wheat.pw.usda.gov/ITMI/Repeats/ TREP_3153	*Triticeae*	>>
Inga	http://wheat.pw.usda.gov/ITMI/Repeats/ TREP_1527	*Hordeum vulgare*	>>
Usier	http://wheat.pw.usda.gov/ITMI/Repeats/ TREP_704	*Hordeum vulgare*	>>
ATCOPIA43	AT1TE43975	*Arabidopsis thaliana*	>>
Osr7	retrieved manually from BAC AP002538	*Oryza sativa*	>>
Osr8	retrieved manually from BAC AC021891	*Oryza sativa*	>>
Tnd-1	AF059674	*Nicotiana debneyi*	>>
OPIE-2	U68408	*Zea mays*	>>
PREM-2	U41000	*Zea mays*	>>
Tst1	X52387	*Solanum tuberosum*	Pseudovirus
Hopscotch	U12626	*Zea mays*	>>
Stonor	AF082134	*Zea mays*	>>
Osr1	AB046118	*Oryza sativa*	>>
Tto1	D83003	*Nicotiana tabacum*	>>
Tnt1	X13777	*Nicotiana tabacum*	>>
CIRE1	AM040263	*Citrus sinensis*	>>
AtRE1	AB021263	*Arabidopsis thaliana*	>>
RIRE1	D85597	*Oryza australiensis*	>>
PDR1	X66399	*Pisum sativum*	>>
Tpv2	AJ005762	*Phaseolus vulgaris*	>>
Fourf	AAK73108	*Zea mays*	>>
Melmoth	Y12321	*Brassica oleracea*	>>
OARE-1	AB061327	*Avena sativa*	>>
Art1	Y08010	*Arabidopsis thaliana*	>>
BARE-1	Z17327	*Hordeum vulgare*	>>
Evelknievel	AF039373	*Arabidopsis thaliana*	>>
Panzee	AJ000893	*Cajanus cajan*	>>
Retrofit	U72726	*Oryza longistaminata*	>>
Ta1-3	X13291	*Arabidopsis thaliana*	>>
Tgmr	U96748	*Glycine max*	>>
Ty1	M18706	*Saccharomyces cerevisiae*	>>
Ty4	M94164	*Saccharomyces cerevisiae*	>>
AtRE2	AB021264	*Arabidopsis thaliana*	>>
Wis2	CT009735	*Triticum aestivum*	>>
Retrosor4	AF061282	*Sorghum bicolor*	>>
Copia	X04456	*Drosophila melanogaster*	Hemivirus
Moscopia	AF134899	*Aedes aegypti*	>>
Osser	X69552	*Volvox carteri*	>>
Tca5	AF065434	*Candida albicans*	>>
