# Supplementary material for: Highly conserved motifs in non-coding regions of Sirevirus retrotransposons: the key for their pattern of distribution within and across plants?
Source: BMC Genomics. 2010 Feb 4;11:89. doi: 10.1186/1471-2164-11-89 (PMC2829016; doi:10.1186/1471-2164-11-89)
Supplement: Additional file 2 — Phylogenetic and structural domain analysis of the Sirevirus and classic Ty1/copia retrotransposons. This file contains supplementary Figure S1 showing the phylogenetic analysis of the Ty1/copia retrotransposons based on the RT/RH domains, supplementary Figures S2 and S3 with alignments of the IR arms of each Sirevirus and classic retrotransposon respectively, supplementary Figure S4 showing the distribution of the novel Sirevirus RM boxes, and supplementary Figure S5 with the 5'LTR alignment of the OPIE-2 and PREM-2 Sireviruses. [file 1471-2164-11-89-S2.PDF]

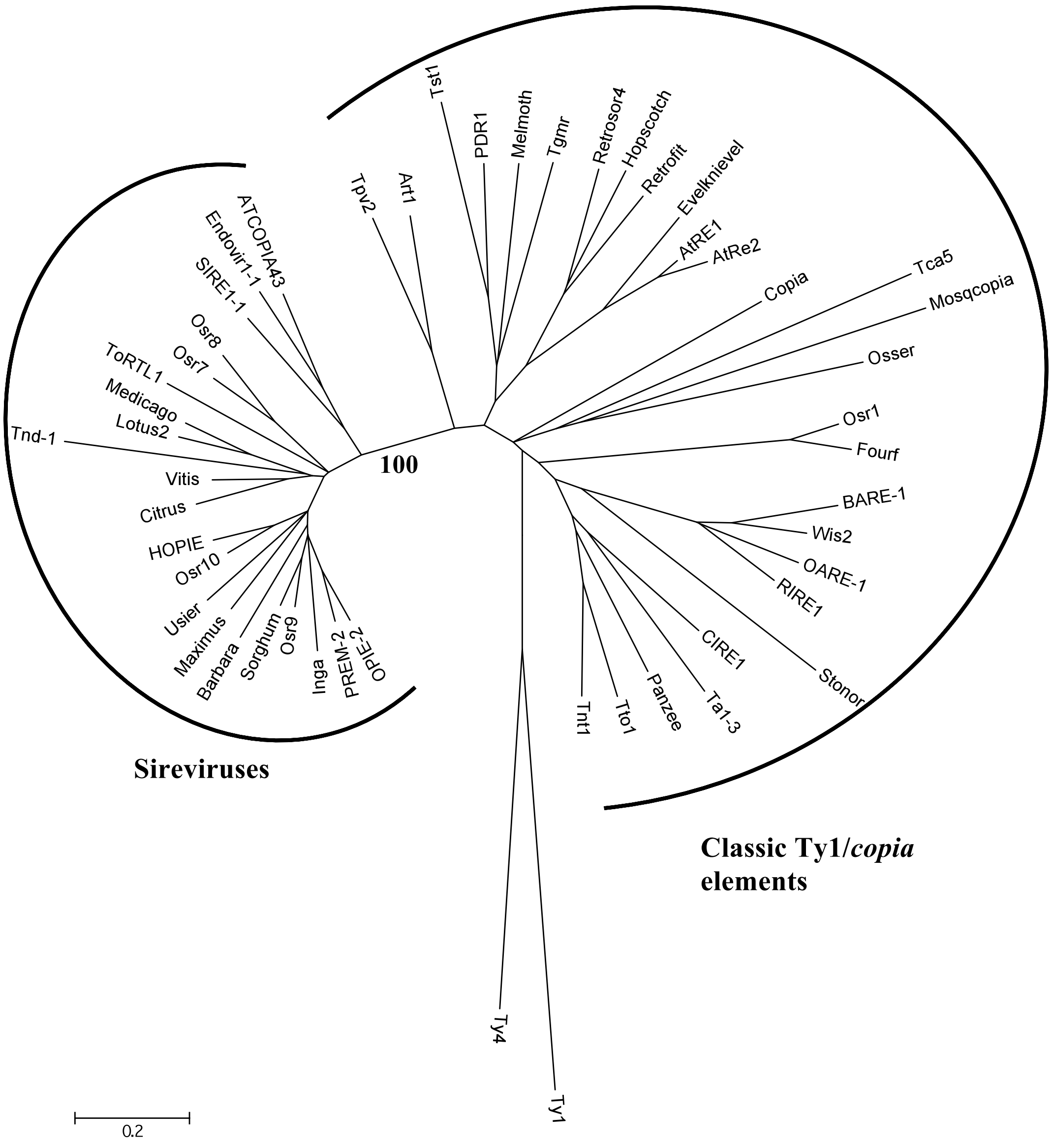

Figure S1. Phylogenetic analysis of the *Pseudoviridae* dataset based on the *RT*/*RH* domain. The Sirevirus genus forms a separate branch, which is supported with 100% confidence value by the bootstrap analysis.

Element	Left arm/right arm	Length of the arms and % identity
Osr10	5′-TTGTCATCAATTACCAAAA-PPT/3′LTR<br>TTGTCATTAATTACCAAAA<br>******* ***********	19-19 94%
Osr9	5′-TCTAATCGTATTGTCATCAATCACCAAAA-PPT/3′LTR<br>TCTAATCGCATTGTAATTAATCACCAAAA<br>******** ***** ** ***********	29-29 90%
Osr7	5′-TTTTTGC----TTGGGA-TTGTCAATGTTCCCATCA-PPT/3′LTR<br>TTCTTGCACTTCCGGTACTTGTCAAT-TTCCCATCA<br>** **** ** * ******** *********	31-35 83%
Sorghum	5′-AGCTC--TAATTTGGTTGTCATCAATCACCAAAA-PPT/3′LTR<br>AGTTCCCTAATTCGGTGGTCATCAA-CGCCAAAA<br>** ** ***** *** ******** * ******	32-33 84%
HOPIE	5′-GTTGTCATCAATTACCAAAA-PPT/3'LTR<br>GTTGTCATTAATTACCAAAA<br>******** ***********	20-20 95%
SIRE1-1	5′-TGTCTAAGT----ATGTTTTAGACAAAATTT--GCCAA-PPT/3′LTR<br>TGTCTAAGTGCTTATGTTTTA-ACAAAATTTTAGCCAA<br>********* ******** ********* *****	32-37 96%
OPIE-2	5′-TTTATG---TTGTGTTGGCA-TAAATCACCAAAA-PPT/3′LTR<br>TTTAAGCACTTGTGTTTGCACTCAATCACCAAAA<br>**** * ******* *** * ***********	30-34 90%
PREM-2	5′-TTTGTGTTGG-CA--TCAATCACCAAAA-PPT/3′LTR<br>TTTGTGTTGGGCAATTCAACCACCAAAA<br>********** ** **** ********	25-28 96%
Medicago	5′-AAATACTATTGT-TTGTCATCATCAAAA-PPT/3′LTR<br>AATTTTTAATACCTTGTTATCATCAAAA<br>** * ** * **** **********	27-28 74%
Citrus	5′-AA--TGTGCTTTGTCATCATAAAAA-PPT/3′LTR<br>AACATGTGGTTTGTTATCATCAAAA<br>** **** ***** ***** ****	23-25 86%
Tnd-1	5′-ATCCCGATACAGGTTTGCCA-TTATCAAAA-PPT/3′LTR<br>ATCCTCGT-T-GGTTTGGCATTTATCAAAA<br>**** * ****** ** *********	29-28 78%
ToRTL-1	5′-GTTTGTCA--TCATCAAAA-PPT/3′LTR<br>GTTTGTCAAATCATCAAAA<br>******** *********	17-19 100%
Vitis	5′-TAACCACAAGGTTTGTCATCATCAAAA-PPT/3′LTR<br>TAACCAC--GGTTTGTTATCATCAAAA<br>******* ******* **********	27-25 96%
Lotus2	5′-AATTGTTCATCAAAAATCTTAGTTTTGTCATCATCAAAA-PPT/3′LTR<br>AATTGTTCATATCAAAAGGTTAACTTGTAATCATCAAAA<br>********** *** * **** **********	39-39 71%
Barbara	5′-TGATCTATATGTTGTCATCAATTACCAAAA-PPT/3′LTR<br>TGAGCTATATGTTGTTATGAATTACCAAAA<br>*** *********** ** ***********	30-30 90%
Maximus	5′-ACTTAACCTAATTGTCATCAA-CCACCAAAA-PPT/3′LTR<br>ACTTAACCTATAAGTCTTCAAACCACCAAAA<br>********** *** **** *********	30-31 86%
Inga	5′-CGGTATTGTCATCAATCCACCAAAA-PPT/3′LTR<br>CGGTACTGTCATCAATC-ACCAAAA<br>***** *********** *******	25-24 95%
Usier	5′-CTATGTATATGTTGTCATCAACTACCAAAA-PPT/3′LTR<br>CTATGTATGTGTTGTCATTAAC-ACCAAAA<br>******** ********* *** *******	30-29 93%

Figure S2. Alignment of the left and right IR arms of each Sirevirus. The reverse complement of the right arm was used for the analysis.

Element	Left arm/right arm	Length of the arms and % identity
Osr1	5'-ATGAGCCTTGAAA---ATTTG-PPT/3'LTR<br>ATGAGCCTTGGAATTTATTTG<br>********** ** *****	18-21 94%
Tnt1	5'-CTACCTCCTCTGGATGAATGAGACTGG-PPT/3'LTR<br>CTAATACTTCT---TCAATGAGA-TGG<br>*** * *** * ******* ***	27-23 78%
Copia	5'-ACGTTATTTTTG-PPT/3'LTR<br>ACGTTATTTTTG<br>************	12-12 100%
CIRE1	5'-ATCAACTGAA-TCAA-CTGCA-PPT/3'LTR<br>ATCAACTGAAGTCAATCTGCA<br>********** **** *****	19-21 100%
RIRE1	5'-CATGATTGACTCTAGTGCA-PPT/3'LTR<br>CATG-TTGCCTCTAGGGCA<br>**** *** ****** ***	19-18 88%
Tpv2	5'-ACATTTAAGTTTAT-PPT/3'LTR<br>ACATTAAAGTTTAT<br>***** ********	14-14 92%
Fourf	5'-ATACTTCATCATTACAAACCCTAGAGTTT-PPT/3'LTR<br>ATTCTCCACCATTACACACCATAGAGTTT<br>** ** ** ******* *** ********	29-29 82%
OARE-1	5'-ATTATTGACTCTAGTGCA-PPT/3'LTR<br>ATTATTGCCTCTAGGGCA<br>******* ****** ***	18-18 88%
Art1	5'-CAAAATCAAGTTTA-PPT/3'LTR<br>CAAAATCAAGTTTA<br>**************	14-14 100%
BARE-1	5'-ATTATTGACTCTAGTGCA-PPT/3'LTR<br>ATCATTGCCTCTAGGGCA<br>** **** ****** ***	18-18 83%
Moscopia	5'-AGTGAAGCAGGTAACCGCTG-PPT/3'LTR<br>AGTGAAGGGGTACACCGATG<br>******* * **** **	20-20 70%
Osser	5'-CAGTTC--CCAGAGGTCTGCCCGC-PPT/3'LTR<br>CAGTTCGTCCAGAGAGCTGACCGC<br>****** ****** *** ****	22-24 86%
Ta1-3	5'-TT-GGAATAGGATCA-PPT/3'LTR<br>TTAGGAATTGGATCA<br>** ***** ******	14-15 92%
Wis2	5'-ATTATTGACTCTAGTGCA-PPT/3'LTR<br>ATTATTGCCTCTAGGGCA<br>******* ****** ***	18-18 89%

Figure S3. Alignment of the left and right IR arms of each classic retrotransposon. The reverse complement of the right arm was used for the analysis.

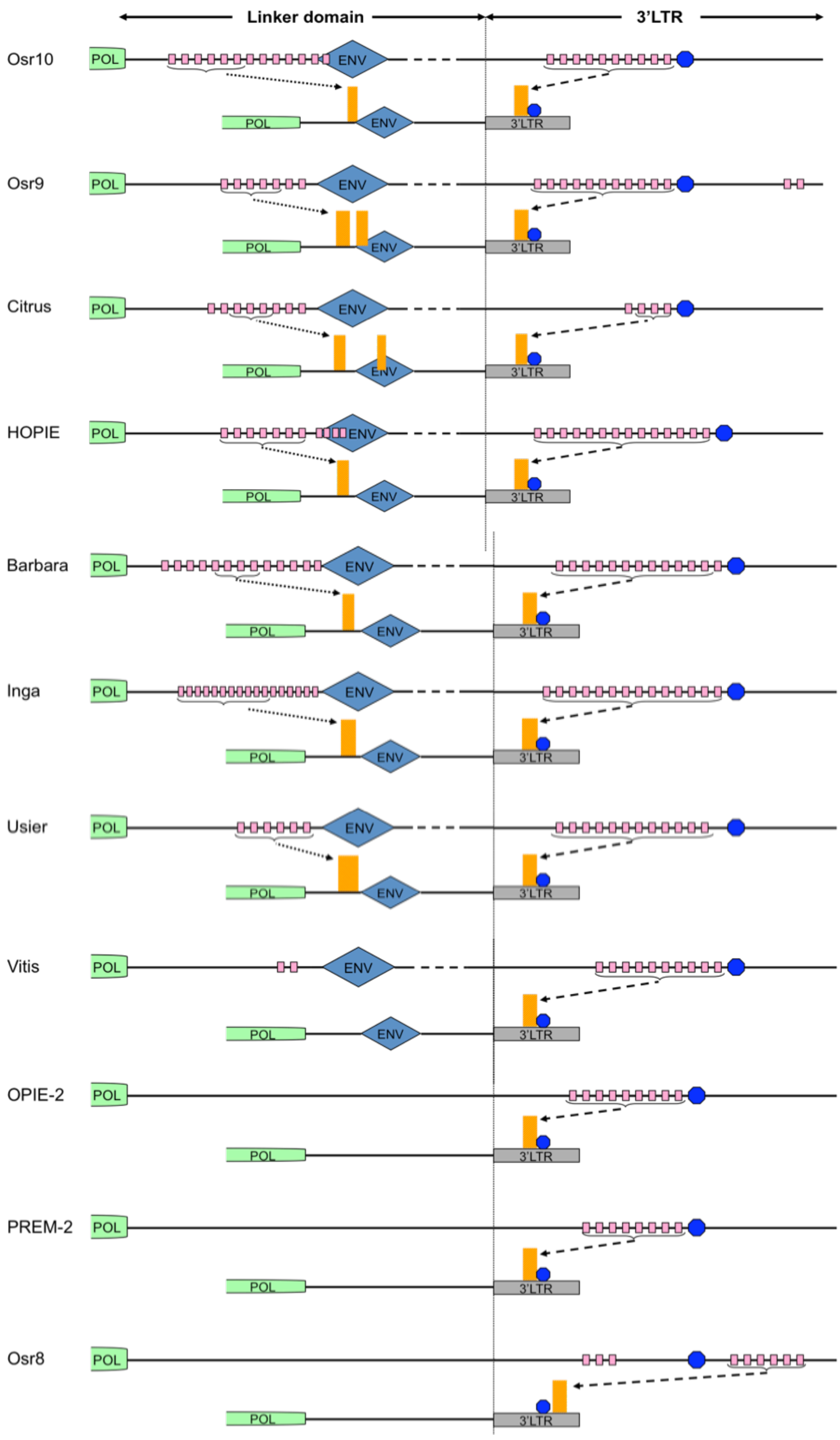

Linker domain
3'LTR
Osr10
Osr9
Citrus
HOPIE
Barbara
Inga
Usier
Vitis
OPIE-2
PREM-2
Osr8
POL
ENV
3'LTR

Figure S4. Organization of the Sirevirus RM boxes (pink) at the 5' side of the *ENV* gene and upstream the TATA box (blue circle) in the 3'LTR. All or a section of the RM clusters define the borders of CpG islands (orange bar) (see also fig. 4b).

```
CLUSTAL 2.0.5 multiple sequence alignment

PREM-2          TGAAAGGGAATTAGGCTTACACCTAGTTCCTAAATAATTTTGGTGGTTGAATTGCCCAAC
OPIE-2          TGAAAGGGAAATGTGCCTTTGGGCCATTTCTAAGTA-TTTTGGTGATTGAGTG--CAAAC
                ********** *  ** *        ** **** ** ******** **** *   * ***

PREM-2          ACAAATCTTTGGACTAACTAGTTTGCCCAAGTGTATAGATTATACAGGTGTTAAAGGTTC
OPIE-2          ACAAGTGCTTAAATGTGAAAATGTGCCCGTG-------GTTGAACA--------AAGTGC
                **** *  **  *      * * *****  *        **  ***        * ** *

PREM-2          ACACTCAGCCAATTAAAAGACCAAGTTTTGGATTCAACAAAGGAGCAAAAGGGGCAACCG
OPIE-2          AAATCAA---AATTAAAGGTATGTTTCTAAGCCTTAGTACATTGGT--------------
                * *   *   ******* *      * *  *  * *  * *   *

PREM-2          AAGGCACCCCTGGTCTGGCACCGGACTGTCCGGTGCGCCACCGGACATGTCCGGTGCACC
OPIE-2          ------TTTGTGTACTAATACC---TTGTCTAA-GTGTTA---GAAACAGGAAGAAGAAT
                          **  **   ***    ****    * *  *   ** *      *   *

PREM-2          AGGGGGACTCAGACTCAAACTCACCACCTTCGGGAATTCACAGGGTCGACTCGGCTATAA
OPIE-2          AAAAGAAAAGAAGTGGAGAGTGGCTGTGTACAGCCA-----AAGGCTGTTTCGGGC-TGG
                *   * *   *     * * *  *    * * *  *     * **  *  ****   *

PREM-2          TTCACCGGACTGTCCGGTGTACACCGGACAGTGTCCG-TGCGCCAAGGGAGGTCGGCCTC
OPIE-2          GGCACCGGACTGTCCGGTGTGCACCGGACAGTGTCCGGTGCGCCAGACCAGCGCGG----
                  ****************** **************** *******    **  ***

PREM-2          AGGAACTCGCCAGCTTCGGGAAAAGCCAACGGCTCGTGCA-CTATAATTCACCGGACTGT
OPIE-2          AGCAAACCAGCCGCTCTCGGGTTTTTCTCCGGCGACTTCGGCTAAAATTCACCGGACTGT
                ** **  *  * ***   **      *  ****   * *  *** ***************

PREM-2          CCGGTGTGCACCGGACTGTCCGGTGCGACTCCGGAGCAACGGTCATCTCCGCGCCAACGG
OPIE-2          CCGGTGTGCACCGGACTGTCCGGTGAGCC--------AACGGTCGGCT--GGGCCAACGG
                ************************* * *        *******  **  * ********

PREM-2          CTCTCTGCCGCGCATTTAATGCGCTCTGCGCGTGCAGGAGTCAGAATCGCCCATGCTAGC
OPIE-2          ---TCGGCCGCGCGATTG--GCGCGCGACACGTGGCCGAGCCAA---CGGTCGGAAAGGT
                   ** *******  **   **** *  * ****   *** **    **  *      *

PREM-2          ACACCGGACATCAAACAGTACCTGTCCGGTGTGCACCGGACAC-CCAGGCGGGCCCACAA
OPIE-2          ACACCGGAC-------------TGTCCGGTGTGCACCGGACATGTCCGGTGCGCCAACTG
                *********             ********************   * ** * *** **

PREM-2          GTCAGAAG-CTCCAACGGTCAGAATCCAACGGCAGTG------ATGACGTGGCAGGGGGC
OPIE-2          TGCGCAGATCTGCATCAGAAAGCAACGGTCGGATGAGCTTTTTATGGAAACAAATCGGGC
                  *  *   ** ** * *  ** * *   ***  * *      ***       *  ****

PREM-2          ACCGGAC--TGTCCGGTGTGCACCGGACTGTCCGGTGCGCCATCGAAC---------AGA
OPIE-2          ACCGGACAGTGTCCGGTGTGCACCGGACTGTCCGGTGCGCCACGAGACTGAAGGCAAAGA
                *******  *********************************    **         ***

PREM-2          CAGCCTCCCAA---------CGGCCACATTTGGTGG--TTGGGGCTATAAATACC-CCAA
OPIE-2          TGGCCTTCCAGATTTGTTCCCAACGGCTCCTAGCTGCCTTGGGGCTATAAAAGGGACCCC
                  **** ***          *  *  *   * *  *  *************     **

PREM-2          CCACCCCACCATTCATTGCATCCAAGTTTTCTCAC----TTCTCAACCACTTACA----A
OPIE-2          TTGGCGCATGGAGGAGTACCCCAAAGCATTCCTACAACACTTCTAAGCACCAAGACGTCA
                    * **      * * *  * ***  ***  **     *   ** ***  * *    *

PREM-2          ----GAGCTAGGCATTCAATTCTAGACACATTCAAAGAGATCA-AATCCTCTCCAATTCC
OPIE-2          ATCTCACGCGTTCGTTTCATTGTGATAGCATATAGAGCTCTTGTGGAGTTTTTGAGTTGT
                     *      * **  *** *     ***  * **   *        * *  * **

PREM-2          ACACAAAACCCTA---GTGACTAGTGAGAGTGAT-TTGCCGTGTTCATTTGAGCTCTTGC
OPIE-2          GTTGCGAGCTCTTTGTGCGACTTGTGTGCGTGTTGTTGCTCTGATCTTTTGAAGTCTTGT
                      * * **    * **** *** * *** * ****  ** ** *****  *****

PREM-2          GCTTGGATTGCTTCTTTTCTTTCTCACTTGTTCTTGTGATCAAAACTCCATTGTAATCAA
OPIE-2          G--TGCGTTGCT-CATTCCCTCCTTACTCCGTGATTCTTTGTGAACATCAAAGTG-TAAG
                *  **  ***** * ** * * ** ***   *  *    *   ***  **  **  * *

PREM-2          GGCAAGAGGCACCAATTGTGTGGTGGCCCTTGCGGGGAAGTTTTGTTCCCGGCTTTGATT
OPIE-2          GGCGAGAGGCTCCAAGT-TGTGGAGATTCCT-CGCAAACG----------GGATAAGAAA
                *** ****** **** * ***** *   * * **   * *          ** *  **

PREM-2          TGAGAAGAGAAGCTCACTCG-GTCCGAGT-GACCGTTTGAGAGA--GGGAAGGGTTGAAA
OPIE-2          AGAAAAGCATAACACTGTGGTATTCAAGTTGATCATTGGATCACTTGAGAGGAGTTGAGT
                 ** ***   * * *  * *  * * *** ** * ** **      * ** * *****

PREM-2          GAGACCCG-GCCTTTGTGGCCTCCTCAACGGGGAGTAGG------TTTG-AGAGAACCGA
OPIE-2          GCAACTCTCGTCCGTTGGGACGCCACAACGTGGAGTAGGCAAGTTTTTGTACTTGGCCGA
                *  ** *  * *  *  ** * ** ***** ********      **** *     ****

PREM-2          ACCTCGGTAAAACAAATCCGTGTGTCACACTTCATTATTTGCTCGCGATT--TGTTTTGC
OPIE-2          ACCACGGGATAA-ACCACTGTGTCTTCTCTGTGTTTGATCTTCTGTGGTTATCGTATTGT
                *** *** * ** *   * **** *      *  **  *     * * **   ** ***

PREM-2          GCC---CTCTCTCGCGGACTCGTTTATATTTCTAACACTAACCCGGCTTGTAGTTGTGT-
OPIE-2          GCAAGGCCTTCACTCTAGCCACTTGGCATTAACTGTGCTAACTCTTAATCAAGTTTTGTG
                **    *  ** * *   *   **   ***       ***** *    *  **** ***

PREM-2          -TTATATTTGTAAATTT--CAGTTTCGCCCTATTCACCCCCCC-TCTAGGCGACTATCA
OPIE-2          GCTATAAGTTTAAGTTTTACAGGATCAC-CTATTCACCCCCCCCTCTAGGTG-CTCTCA
                  ****  * *** ***  ***  ** * ************** ****** * ** ***
```

Figure S5. Alignment of the 5'LTRs of OPIE-2 and PREM-2. The herein introduced motifs are the only highly similar long segments amidst diverse sequences. The RM boxes are shown in red, the TATA box domains and the polyC-rich integrase signal are shaded in grey, the right IR arms in green and the beginning of the LTRs in black.
