# Supplementary material for: Highly conserved motifs in non-coding regions of Sirevirus retrotransposons: the key for their pattern of distribution within and across plants?
Source: BMC Genomics. 2010 Feb 4;11:89. doi: 10.1186/1471-2164-11-89 (PMC2829016; doi:10.1186/1471-2164-11-89)
Supplement: Additional file 3 — The stress-related DNA binding factors that target core sites within the RMs. This file contains supplementary Table S2 with information related to the binding site and its orientation within each RM and the stress nature of the DBFs. [file 1471-2164-11-89-S3.PDF]

Table S2. **Stress-related DNA binding factors that target core sites within the RMs**

Element	RM†	DNA binding factor	Orientation	Comment
**Vitis**	CGGTCGACCG	C-repeat/dehydration responsive element (CRT/DRE)	plus/minus	imparts responsiveness to cold, dehydration and high salinity (osmotic) stresses [1]
**Osr10**	CGGTCTGACCG	W box	plus	found in the promoter of many defense genes, mediates response to pathogen infection, pathogen-derived elicitors and wounding [2, 3]
**Osr8**	CGGTCAGACCG	W box	minus	>>
**Usier**	GTCGGACGTCCGAC	Abscisic acid responsive elements (ABREs)	plus/minus	found in the promoter of many defense genes that are induced by a plethora of environmental stresses [4, 5]
**HOPIE**	CGGACCGTCCG	Phenylalanine ammonia lyase (PAL) genes	plus	core hexamer lies in the promoter of PAL genes involved in response to a/biotic stresses such as fungal elicitors and wounding [6, 7]
**Barbara**	TCGGTCYCACCGA	Auxin response factor (ARF)	minus	ARF binding site found within the sulfur deficient responsive element (SURE) of the sulfate transporter *SULTR1* gene in *Arabidopsis* roots [8]
**Inga**	AGCGGTASTACCGCT	Copper and hypoxia response element	plus/minus	core tetramer found in the *Cyc6* and *Cpx1* gene of the *Chlamydomonas* green alga [9]
**Sorghum**	AGGGCGGCAGTGCCGCCCT	GCC-box element	plus/minus	*cis*-acting GCC-box lies in the promoter of many fungal and bacterial pathogen-responsive genes [10]
**Osr9**	TTTTCGGACWTRTCCGAAAA	Low temperature responsive element (LTRE)	plus/minus	regulates cold-inducible gene expression in barley [11]

† The underlined nucleotides mark the putative core recognition sites within the RMs.

References

1. Thomashow MF: **So what's new in the field of plant cold acclimation? Lots!** *Plant Physiol* 2001, **125**(1):89-93.
2. Eulgem T, Rushton PJ, Robatzek S, Somssich IE: **The WRKY superfamily of plant transcription factors**. *Trends in Plant Science* 2000, **5**(5):199-206.
3. Nishiuchi T, Shinshi H, Suzuki K: **Rapid and transient activation of transcription of the ERF3 gene by wounding in tobacco leaves - Possible involvement of NtWRKYs and autorepression**. *J Biol Chem* 2004, **279**(53):55355-55361.
4. Busk PK, Pages M: **Regulation of abscisic acid-induced transcription**. *Plant Molecular Biology* 1998, **37**(3):425-435.
5. Foster R, Izawa T, Chua NH: **Plant Bzip Proteins Gather at Acgt Elements**. *Faseb Journal* 1994, **8**(2):192-200.
6. Dixon RA, Paiva NL: **Stress-Induced Phenylpropanoid Metabolism**. *Plant Cell* 1995, **7**(7):1085-1097.
7. Logemann E, Parniske M, Hahlbrock K: **Modes of Expression and Common Structural Features of the Complete Phenylalanine Ammonia-Lyase Gene Family in Parsley**. *Proc Natl Acad Sci U S A* 1995, **92**(13):5905-5909.
8. Maruyama-Nakashita A, Nakamura Y, Watanabe-Takahashi A, Inoue E, Yamaya T, Takahashi H: **Identification of a novel cis-acting element conferring sulfur deficiency response in Arabidopsis roots**. *Plant J* 2005, **42**(3):305-314.
9. Kropat J, Tottey S, Birkenbihl RP, Depege N, Huijser P, Merchant S: **A regulator of nutritional copper signaling in Chlamydomonas is an SBP domain protein that recognizes the GTAC core of copper response element**. *Proc Natl Acad Sci U S A* 2005, **102**(51):18730-18735.
10. Hao DY, Ohme-Takagi M, Sarai A: **Unique mode of GCC box recognition by the DNA-binding domain of ethylene-responsive element-binding factor (ERF domain) in plant**. *J Biol Chem* 1998, **273**(41):26857-26861.
11. Dunn MA, White AJ, Vural S, Hughes MA: **Identification of promoter elements in a low-temperature-responsive gene (blt4.9) from barley (Hordeum vulgare L.)**. *Plant Molecular Biology* 1998, **38**(4):551-564.
