# Supplementary material for: Highly conserved motifs in non-coding regions of Sirevirus retrotransposons: the key for their pattern of distribution within and across plants?
Source: BMC Genomics. 2010 Feb 4;11:89. doi: 10.1186/1471-2164-11-89 (PMC2829016; doi:10.1186/1471-2164-11-89)
Supplement: Additional file 4 — List of the RT/RH peptide sequences that were used for the construction of the Ty1/copia phylogenetic tree. [file 1471-2164-11-89-S4.PDF]

The *RT*/*RH* protein sequences of the Sirevirus and classic retrotransposon datasets that were used in the phylogenetic analysis

```
>Lotus2
YVDDIIFGSANQSLCKEFSEMMQAEFEMSMMGELKYFLGIQVDQTPEGTYIHQSKYTKELLKKFNMLESTVAKTPMHPTCILEKEDISG
KVCQKLYRGMIGSLLYLTASRPDILFSVHLCARFQSDPRETHLTAVKRILRYLKGTTNLGLMYKKTSEYKLSGYCDADYAGDRTERKST
SGNCQFLGSNLVSWASKRQSTIALSTAEAEYISAAICSTQMLWMKHQLEDYQILESNIPIYCDNTAAISLSKNPILHSRAKHIEVKYHF
IRDYVQKGVLLLKFVDTDHQWADIFTKPLAEDRFNFILKNLNMDFCPE
>Osr10
YVDDIIFGSTNEVFCKEFGDMMSREFEMSMIGELSFFLGLQIKQLKDGTFVSQTKYIKDLLKRFGLEDAKPIKTPMATNGHLDLDEGGK
PVDLKLYRSMIGSLLYLTASRPDIMFSVCMCARFQAAPKECHLVAVKRILRYLKHSSTIGLWYPKGAKFKLVGYSDSDYAGCKVDRKST
SGSCQMLGRSLVSWSSKKQNSVALSTAEAEYVSAGSCCAQLLWMKQTLLDYGISFTKTPLLCDNDSAIKIANNPVQHSRTKHIDIRHHF
LRDHVAKCDIVISHIRTEDQLADIFTKPLDETRFCKLRNELNVIDFSNVA
>Osr9
YVDDIIFGSTNKSFSEEFSRMMTKRFEMSMMGELKFFLGLQIKQLKEGTFICQTKYLKDMLKKFGMENAKPIHTPMPSNGHLDLNEQGK
DVDQKVYRSIIGSLLYLCASRPDIMLSVCMCARFQAAPKECHLVAVKRILRYLVHTPNLGLWYPKGARFDLIGYADADYAGCKVDRKST
SGTCQFLGRSLVSWSSKKQNSVALSTAEAEYVSAGSCCAQLLWMKQTLRDYGLNVSKIPLLCDNESAIKIANNPVQHSRTKHIDIRHHF
LRDHSTRGDIDIQHVRTDKQLADIFTKPLDEARFCELRSELNILDSRNVA
>Osr7
YVDDIIFGGSSHALVAQFSDVMSREFEMSMMGELTFFLGLQIKQTKEGIFVHQTKYSKELLKKFDMADCKPIATPMATTSSLGPDEDGE
EVDQREYRSMIGSLLYLTASRPDIHFSVCLCARFQASPRTSHRQAVKRIFRYIKSTLEYGIWYSCSSALSVRAFSDADFAGCKIDRKST
SGTCHFLGTSLVSWSSRKQSSVAQSTAEAEYVAAASACSQVLWMISTLKDYGLSFSGVPLLCDNTSAINIAKNPVQHSRTKHIEIRYHF
LRDNVEKGTIVLEFVESEKQLADIFTKPLDRSRFEFLRSELGVIHPYGLI
>Osr8
YVDDIIFGYSTHALVVDFAENMRREFEMSMMGELSYFLGLQIKQTPQGTFVHQTKYTKDLLERFKMENCKPISTPIGSTAVLDPDEDGE
AVDQKEYASRPDIQFVVCLCARFQASPRASHRQAVKRIMRYLNHTLEFGIWYSTSSSICLSGYSDADFGGCRIDRKSTSGTCHFLSTSL
IAWSSRKQSSVAQSTAESEYGAAASCCSQILWLLSTLKDYGLTFEKVPLLCDNTSAINIAKNPVQHSRTKHIDIHFHFLRDHVEKGDVE
LQFLDTKLQIADIFTKLLDSNRFAFLRGELGIIHPFGMV
>Sorghum
YVDDIIFGSTNQVFCEEFSRIMTKRFEMSMMGELKYFLGFQIKQLKEGTFISQTKYTLDMLKKFDMEKAKPINTPMPTNGHLDLDDKGK
AVDIKVYRSMIGSLLYLCASRPDIMLSVCMCARFQANPKECHLVAVKRILRYLVHTPNLGLWYPKGSKFNLLGYSDSDYAGCKVDRKST
SGTCQFLGRSLVSWSSKKQNCVALSTAEAEYIAAGACCAQLLWMRQTLRDFGCQFTKIPLLCDNESAIKLANNPVSHSRTKHIDVRHHF
LRDHEAKGDIEIRHVSTEKQLADIFTKPLDETRFCALRSELNILDSRNVV
>Medicago
YVDDIIFGSTNASLCKEFSKLMQDEFEMSMMGELKFFLGIQINQSKEGVYVHQTKYSKELLKKFKLEDCKVMNTPMHPTCTLNKEDTGT
VVDQKLYRGMIGSLLYLTASRPDILFSVCLCARFQADPRESHLTAVKRIFRYLKGTTNLGLLYRKSLDYKLIGFCDADYAGDRIERKST
SGNCQFLGENLISWASKRQATIAMSTAEAEYISAASCCTQLLWMKHQLEDYQINANSIPIYCDNTTAICLSKNPILHSRAKHIEIKHHF
IRDYVQKGILDIQFIDTEHQWADIFTKPLYVERFDFIKKNLNMHFVSN
>Citrus
YVDDIIFGFTNELLCKDFSSCMSQEFEMSMMGELKYFLGLQIKQNEEGIFINQAKYVRDLLKRFGYDNRTAKSTPMSTTIKLDKAEKGK
EVDIKTYRGMIGSLLYLTASRPDIMFSVCLCARFQSCPKESHMLAVKHIFRYLIGTINLGLWYPRGTHIDLTCYSDADFAGYKVDRKST
SGTCHFLGHYLVSWFSKKQNSVALSTTEAEYIAAGSCCAQILWMKQTLRDYGIKLDQIPILCDNTSAINLSKNPIQHSRTKHIEIRHHF
LRDHVQKGDVMIKFVSTENQLADIFTKPLCEEHFIKIRHELGMMNVES
>HOPIE
YVDDIIFGSTNELFCEEFGKMMSKEFEMSMIGELSFFLGLQIKQLKDGIFISQSKYLKDMLKKFGLENAKPIKTPMATNGHLDLDEGGT
MVDQKLYRSIIGSLLYITASRPDVMFSVCMCARFQASPREIHLKAAKRILRYLKYTPNIGLWYPKGAHFELIGYSDSDYAGCKVDRKST
SGCCQFLGRSLVSWSSKKQNSVALSTAEAEYISAENCCAQLLWMKQTLLDYGIIFKNVPFMCDNESAVKLATNPVQHSRTKHIDIRHHF
LRDHVGKGDFSIYSIGTDDQLADIFTKPLDETRFCSLRSEMNVIDLSNVA
>Tnd-1
YLDDIFFGTTNMFVCKEFVRLITSQFKMSMMDERKLILKLQVKQMKEGTFICQTKYCKELLN*LEISNTKAMATSMNMTILDRNERGKE
VNTKIYRGMIRSLLYFTASRLDIIFSVY*YARYESCPKESRLNAAK*SILLEILNVGLCYPKDSSCQSLGYSDSNFARCKLDRKSIIGT
CHLLGNSLVSWISKKRVNVALSATEAEYTATGSCCSQILWMK*QLLDYGLSLDYVPLRCDNTSTISLTKN*ILHSRPKPIKIRHHFMKD
HVLKGDVQIEFVITKHQFDDIFTRPLDKESFFRILSELGILDS
>ToRTL1
YVDDIISGTTSEHLCEEFSSLMGREFEMSMMGELTFFLGLQIKQSSNGTSICQEKYIKELLKKFNMFDSKPIDTSMGTNPKMIVEKSDP
LVNQAMYREIIGSLLYLTASRPDIVYSVGICARFQACPRDSHLKAAKRILRYLKKIGNLVLFYPAGDTFDLVGFADADFAGYQVDRKST
SGMAHFLGSSLISWGTKKQNSVALSTAEAEYVAAAACCSQLLWIRQHLEDFGIHIKAFLLMCDNTSAVSMGKNSFHHKRTKHIDVRHHF
LRDHVEKGNIVLTYCPTEEQIADIFTKALSKDQFERNRLKLGMLISK
>SIRE1-1
YVDDIVFGGMSNEMLRHFVQQMQSEFEMSLVGELTYFLGLQVKQMEDSIFLSQSKYAKNIVKKFGMENASHKRTPAPTHLKLSKDEAGT
SVDQSLYRSMIGSLLYLTASRPDITYAVGGCARYQANPKISHLNQVKRILKYVNGTSDYGIMYCHCSDSMLVGYCDADWAGSVDDRKST
FGGCFYLGTNFISWFSKKQNCVSLSTAEAEYIAAGSSCSQLVWMKQMLKEYNVEQDVMTLYCDNLSAINISKNPVQHSRTKHIDIRHHY
IRDLVDDKVITLEHVDTEEQIADIFTKALDANQFEKLRGKLGICLLEDL
>Endovir1-1
YVDDIVFGGTSDKLVKTFVKTMTTEFRMSMVGELKYFLGLQINQTDEGITISQSTYAQNLVKRFGMCSSKPAPTPMSTTTKLFKDEKGV
KVDEKLYRGMIGSLLYLTATRPDLCLSVGLCARYQSNPKASHLLAVKRIIKYVSGTINYGLNYTRDTSLVLVGYCDADWGGNLDDRRST
TGGVFFLGSNLISWHSKKQNCVSLSSTQSEYIALGSCCTQLLWMRQMGLDYGMTFPDPLLVKCDNESAIAISKNPVQHSVTKHIAIRHH
FVRELVEEKQITVEHVPTEIQLVDIFTKPLDLNTFVNLQKSLGIGEV
>OPIE-2
YVDDIIFGSTNQKSCEEFSRVMTQKFEMSMMGELNYFLGFQVKQLKDGTFISQTKYTQDLLKRFGMKDAKPAKTPMGTDGHTDLNKGGK
SVDQKAYRSMIGSLLYLCASRPDIMLSVCMCARFQSDPKECHLVAVKRILRYLVATPCFGLWYPKGSTFDLVGYSDSDYAGCKVDRKST
```

```
SGTCQFLGRSLVSWNSKKQTSVALSTAEAEYVAAGQCCAQLLWMRQTLRDFGYNLSKVPLLCDNESAIRMAENPVEHSRTKHIDIRHHF
LRDHQQKGDIEVFHVSTENQLADIFTKPLDEKTFCRLRSELNVLDSRNLD
>PREM-2
YVDDIIFGSTNESTCEEFSRIMTQKFEMSMMGELKYFLGFQVKQLQEGTFISQTKYTQDILSKFGMKDAKPIKTPMGTNGHLDLDTGGK
SVDQKVYRSMIGSLLYLCASRPDIMLSVCMCARFQSDPKESHLTAVKRILRYLAYTPKFGLWYPRGSTFDLIGYSDADWAGCKINRKST
SGTCQFLGRSLVSWASKKQNSVALSTAEAEYIAAGHCCAQLLWMRQTLRDYGYKLTKVPLLCDNESAIKMADNPVEHSRTKHIAIRYHF
LRDHQQKGDIEISYINTKDQLADIFTKPLDEQSFTRLRHELNILDSRNFFC
>Vitis
YVDDIIFXATNDSLCEDFSKCMHSEFEMSMMXELNXFLGLQIKQLKEGTFINQAKYIKDLLKRFNMEEAKVMKTPMSSSIKLDMDEKGK
SIDSTMYRGMIGSLLYLTASRPDIMYSVCLCARFQSCPKESHLSXVKRILRYLKGTMNIGLWYPKGDNFELIXFSDADFAGCRVERXST
SGTCHFLGHSLVSWHSKKQNSVALSTAEAEYIAAGLCCAQILWMKQTLSDFNLSFEHVPIKCDNTSAINISKNPVQHSRTKHIEIRHHF
LRDHAQKGDITLEFVSTKDQLADIFTKPLSEEQFSDIRRQLGVISL
>Barbara
YVDDIIFGSTNPLFSEKFGKLMSEKFEMSMMSELKFFLGLQIKQTKEGTFVSQTKYTKDLFKKFNMQECKGMSTPMPTSGHNDLTKDGE
PVDQKVYRSMIGSLLYLCASRPDIMLSVCMCARYQAAPKDCHLKAVKRIVRYLIHTPNFGIWYPKRSSFDLVGYSDSDYARDKVDRKST
SGTCQFLGRSLVSWSSKKQNSVSLSTAEAEYIAAGSCCAQLLWMTQTLKDYGIYVKHVPLLCDHESAMKIGHNPIQHSRTKHIEVRHHF
IRDHVAKGDINLKHVRTDKKLADIFTKPLDEKVFCRLRGELNIIDASNLE
>Maximus
YVDDIIFGCTDKRYSDEFAYMMSEEYQMSMMGELKFFLGLQIRQQRNGIFISQEKYLKDVLRKFDMHECKGAKTPMPTNGHLGTDENGK
DFDQQVYRSMIGSLLYLCASRPDIMLSVCMCACFQAKPKESHHKAVKHILRYLAHTPTLGLWYPKGSNLHLVGYSDSDYAGDRVDRKST
SGTCHFLGRSLVCWSSKKQNCVSLSTAEAEYIAAGSCCAQLLWMKQTLKDYGINMKNVPLYCDNESAIKIAYNPVQHSKTKHIQIRHHF
LRDHVLKGNILIDHVKTDDQLADIFTKPLDEKRFCKLRCELNILESSNVL
>Inga
YVDDIIFGSSNISFNEEFAALMTEKFEMSMMGELKFFLGFEIKQGLEGTFIKQAKYTQDMLKRSELKDVKPVKFPMPTRCKLDSDPNGK
GVDQKVYRSMIGSLLYLCASRPDIMLSVGICARFQSAPKESHYMAVKRIFRYLAHTPNFGLWYPKGANFNLVGYSDSDWAGDCVERKST
SGGCQFLGRSLVSWSSKKQNCVSLSSTEAEYVAAASCCAQLLWMRQTLKDYGVTCDKVPLLCDNQSAIKISLNPVQHSKTKHIDIRHHF
IREHIKLGDIEVHFIHTEEQLADIFTKPLDEARFRELRHELNIIDSSNVD
>Usier
YVDDIIFGSPNIHLCKKFAASMTKTFEMSLNTDLKFFLGFQIQQFQEGIFLSQTKYLKGILENFDMTNAKPMKTPMATDVVLNEDTNGI
PFDPSTYRSMIGSLLYLCASRPNIMLSVGICARFQASPRESHHTAVKHILRYLVHTPKLGLWYPKDAKFDLIGYSDADWAGDKVGRKST
FGACQFLGRSLVSWSSKKQNYVSLSTAEAEYIAAASCATQLLWMRQTLKDYGITYRHVPLLCDNESAIKISENPIDHPRTKHIDIRYHF
LRDHVQKGDIDIAHVGTDMQLTDIFTKPLCVPRFCQLRRELGTLELDNIT
>ATCOPIA43
YVDDIVF*STKQKLVSDFVESMTKEFEMSMVGEMNHFLGLQIKQTDEGVHISQSMYAQGLIQRFGMQTAKTSKTPMSATAKLSADEAGL
SVDEKMYRGMIGSLLYLTASRPDLCFSVGVCARYQANPKQSHLNAMKWILKYVKGTTDVGLFYSKQTNQNLVGFCDADWAGNLDDRRST
TGGCFFLRNNLVSWHSKKQSCVSLSTAEAEYIALGSCCTQLLWMKQMLLDYGMTSNTLLVYCDNMSAINISKNPVQHSRTKHIDIRHHF
IRELVENKIVEISHVSSEKQLADIFTKSLDLNSFLNL
>Tst1
YVDDIILTGDDVVEIKNLKERLASEFEIKDLGPLKYFLGMEVARSKKGIIVSQRKYVLDLLKETGMSGCRPTETPIDPNLKFVKEGKLI
DKGQYQRLVGKLIYLSHTRPDISFAVSLVIQFMHYPREEHQEAVYRILRYLKSSPGKGLFFKKNEQRSLEAYTDADWAGSSIDRRSTSG
YCTFVWGNLVTWRSKKQNVVARSSAEAEYRSMALGICEILWLKRFLEELRRPVSFPMKLYCDNKAAISIAHNPVQHDRTKHVEVTDTSL
KRRLKMEVCAFLLFQQQNKLQIFSQKVFSELLLSPLLAS
>Hopscotch
YVDDIIITGSSPHAIDNVLAKLKDDFAIKDLGDLHYFLGIEVHRKGDGLLLCQEKYARDLLKRVGMECCKPVHTPVATSEKLSASAGTL
LSPEETTKYRSVVGALQYLTLTRPDLSYAINRVCQFLHAPTDLHWTAVKRILRNIQHTIGLGLTIRPSLSLMLSAFSDADWAGCPDDRK
STGGYALFLGPNLISWNSKKQSTVSRSSTEAEYKAMANATAEVIWLQSLLHELGIRLTGIPRLWCDNLGATYLSSKPIFNARTKHIEVD
FHFVRDRVLSKKLDIRLISTNDQVADGFTKALTIGRLNEFRRQSQPRSVVIEGGY
>Stonor
YVDDILLASSDKDLLAETKGFLSSNFDMKDMGEASYVLGIEIRRDRQKRVLGLSQKSYIENVLKRYNMHKCNASPGPIVKGDKFGEYQC
PKNQYEKNKMKSVPYASAIGSIMYAQVCTRPDLAFTTGMLGRYQKNPGIEHWKAVKKALRYLQGTKGLMLTYRRSNSLQIVGYADADWG
GCRDTLKSTSGYVFMLSGGAISWKSCKQTARASSTMHAEFVATYEATGQAIWIKKFVPGLRVVDSIERPLRIYCDNEPAVFFSHNNKSS
GSAKYIDIKCYIVKEKILDHTIQVEHIRTHQMLADPLTKGLPPSVFSKHAAGMGLREYL
>Osr1
YVDDILIFGTSLNVIEEVKDYLSKSFEMKDLGEADVILNIKLQRGDEGGITLVQSHYVDKVLSRFGYSDCKPAPTPYDPSVLLRKNRRI
ARDQLRYSQIIGSLMYLASATRPDISFAVSKLSRFVSNPGDDHWQALERVMRYLKGTMSYGIHYTGYPKVLEGYSDSNWISDADEIKAT
SGYVFTLGGGAVSWKSCKQTILTRSTMEAELTALDTATVEAEWLRELLMDLPVVEKPVPAILMNCDNQTVIIKVNSSKDNMKSSRHIKR
RLKSVRKQKNSGVIALDYVQTARNLADQFTKGLPRNVIDSASREMGLIPT
>Tto1
YVDDMLIVGRNVSRINSLKEQLSKFFAMKDLGPAKQILGMRIMRDREAKKLWLSQEKYIEKVLQRFNMEKTKAVSCPLANHFRLSTKQS
PSTDDERRKMERIPYASAVGSLMYAMVCTRPDIAHAVGVVSRFLSNPGKEHWDAVKWILRYLRGTSKLCLCFGEDNPVLVGYTDADMAG
DVDSRKSTSGYLINFSGGAVSWQSKLQKCVALSTTEAEFIAATEACKELIWMKKFLTELGFSQDGYQLFCDSQSAIHLAKNASFHSRSK
HIDVRYNWIRDVLEKKMLRLEKIHTDENGSDMLTKTLPKGKFEFCREAAGIVDPPYSWKGENC
>Tnt1
YVDDMLIVGKDKGLIAKLKGDLSKSFDMKDLGPAQQILGMKIVRERTSRKLWLSQEKYIERVLERFNMKNAKPVSTPLAGHLKLSKKMC
PTTVEEKGNMAKVPYSSAVGSLMYAMVCTRPDIAHAVGVVSRFLENPGKEHWEAVKWILRYLRGTTGDCLCFGGSDPILKGYTDADMAG
DIDNRKSSTGYLFTFSGGAISWQSKLQKCVALSTTEAEYIAATETGKEMIWLKRFLQELGLHQKEYVVYCDSQSAIDLSKNSMYHARTK
HIDVRYHWIREMVDDESLKVLKISTNENPADMLTKVVPRNKFELCKELVGMHSN
>Copia
YVDDVVIATGDMTRMNNFKRYLMEKFRMTDLNEIKHFIGIRIEMQEDKIYLSQSAYVKKILSKFNMENCNAVSTPLPSKINYELLNSDE
DCNTPCRSLIGCLMYIMLCTRPDLTTAVNILSRYSSKNNSELWQNLKRVLRYLKGTIDMKLIFKKNLAFENKIIGYVDSDWAGSEIDRK
STTGYLFKMFDFNLICWNTKRQNSVAASSTEAEYMALFEAVREALWLKFLLTSINIKLENPIKIYEDNQGCISIANNPSCHKRAKHIDI
KYHFAREQVQNNVICLEYIPTENQLADIFTKPLPAARFVELRDKLGLLQDDQSNAE
>CIRE1
```

YVDDMLIASKYKSEIERLKNLLKAEFEMKDLGNAKRILGMDIIRDRSAGTLFLSQGKYIKKVLERFEMQDCKPVQTPLGPQFKLSAATT
SEDESQMNEFPYAQAVGSLMYAMVCTRSDIAYAVSVVSRYLSCPGKVHWNAVKWIMRYLKGSSTCGLLYGKTKSDKIEVMGFVDSDFAG
DLDRRKSTSRYMFVLNSCLISWKSSLQSGGALSSTEAEFIATTEAVKEAMWLRGLLNELWLNQKIVQVFCDNQNAIHLVKNQMYHERTK
HIDVKLQFIRDEVGKGTVVVSKIHTSVNPADALTKSLPTAKFEFCVNLMGIMPKSN
>AtRE1
YVDDILITGNDPTLLHNTLDNLSQRFSVKDHEELHYFLGIEAKRVPTGLHLSQRRYILDLLARTNMITAKPVTTPMAPSPKLSLYSGTK
LTDPTEYRGIVGSLQYLAFTRPDISYAVNRLSQFMHMPTEEHLQALKRILRYLAGTPNHGIFLKKGNTLSLHAYSDADWAGDKDDYVST
NGYIVYLGHHPISWSSKKQKGVVRSSTEAEYRSVANTSSEMQWICSLLTELGIRLTRPPVIYCDNVGATYLCANPVFHSRMKHIAIDYH
FIRNQVQSGALRVVHVSTHDQLADTLTKPLSRTAFQNFASKIGVTRVPPS
>RIRE1
YVDDILLIGNDIPMLESVKSSLKNSFSMKDLGEAAYILGIRIYRDRSKRLIGLSQSTYIDKVLKRFNMHDSKKGFLPMSHGINLSKNQC
PQTHDERNKMGMVPYASAIGSIMYAMLCTRPDVSYALSATSRYQSDPGEGHWTAVKNILKYLRRTKDMFLVYGGEEDLVVSGYTDASFQ
TDKDDYRSQSGFVFCLNGGAVSWKSSKQDTVADSTTEAEYIAASEAAKEAVWIKKFVSELGVMTSTTGPMSLYCDNSGAIAQAKEPRSH
QKSKHILRRYHLIREIVDRGDVKICKVHTDLNIADPLTKPLPQPKHEAHTRAMGIRYLHD
>PDR1
YVDDIIVTGDDLIERHFLKEKLSAEFEMKDLGQLKYFLRIEVAYSKQGIFISQRKYVLDLLQETGKLGCKPASVPIEQNHRISFEEESD
KVDKGQYQRLVGKLIYLAHTRPDLAYAVSVVSQFMHDPRVRHLQAVDRVLQYLKATPGRGLLFKRGGNLTMETYTDADYAGSVSDRRST
SGYCTFLCGNLVAWKSKKQSVVARSSAEAEFRAMALGICELLWMKQILEDLKIQCEGPMTLFCDNKSAISIAHNPVQHDRTKHIEIDRH
FIKDKLDSGLITTSYVPSRHQLADVLTKDLPTERFRQLTCKLGMIDIDSPA
>Tpv2
YVDDLIIMGNNNEMIEEFKGTMRREFEMTDLGLMKFFLGLEVRQKETGIFVSQETYAKDILKKYKMENCNPVSIPMEPGAKLSKFDGGE
RVDASRYRSLVGSLRYLTCTRPDLSLSVGIISRFMEEPFYSHWKALKRVLRYIQGTISLGLFYSKVEDYKLVGYSDSDWCGEIDDRKST
SGYVFYMGNTTFSWLSKKQPIVTLSTCEAEYVAASWCVCHAIWLRNLLTKMELKQLGATVVQVDNKSAIELAKDPVNHERSKHIDVRFH
FIRDHVKKGSVELVHVASQDQVADIFTKPLPKVFFDKFKKMIGMMDGRNI
>Fourf
YVDDILIFGSNLNVIEEVKNLLSSNFEMKDLGEADVILNIKLVRKADGGVTLLQSHYVEKVLSRFGFSDCDPAPTPYDPSVLLRKNRRI
ARDQLTYSQIIGSLMYLASATRPDISYAVSKLSRFVSKPGDDHWCALERVLRYLKGTMTYGIHYTGNPKVLEGYCDANWISDADELYAT
SGYVFLFGGGAVSWKSCKQTILTKSTMEAELAALDTAGAEAEWLRDFLLDLPVVEKPIPAISMNCDNQTVITKVNSSRNNMKSTRHVKR
RLKSVRKLKNSGVITVDYVHTSNNLADQFTKGLSRNVIESASREMGMRPM
>Melmoth
YVDDIVIASTDDAVAVKLKSDLKSFFKLRDLGSLKYFFLGLEISRTSDGISLCQRKYALELLTSTGMLDCKPSSVPMMPNLKLSKVDGD
LIEDREMYRRLVGRLMYLTITRPDITFAVNKLCQFSSAPRTSHLGAVYKVLQYIKGTVGQGLFYSAHSDLTLKGFADADWAACVDSRRS
TTGFTMFVGDSLISWRSKKQPTVSRSSAEAEYRALALASCEMMWLCILLRELHVASSSVPVLFSDSTAAIYIATNPVFHERTKHIELDC
HTVREKIDKGLLKTLHVRTEDQVADILTKPLFPYQFEHLKSKMCIRNIFCTS
>OARE-1
YVDDILIIGNDINMLNDVKSYLNKCFSMKDLGEAAYILGIKIYRDRQRRLIALSQSTYLDKVLKRFRMDKAKKGSVPMLSGKISSKAQC
LATVNDREAMSKVPYASAIGSIMYAMLCTRPDVSNALSLTSRYQSDPGVEHWTAVKNILKYLNRTKEMFLVYGGDEQLVVKGYVDASFD
TDKDDSKSQKGYVYILNGGAVSWRSCKQSVIAGSIVEAEYMAAAEASSEGVWIRNFITELGMVPSALDPLEILCDNTGAIALAKEPRYH
PKTKHINCKFHIIRDYIADGDLKISKVHTDLNVADPLTKPLPRVKHDQHLSAMGVRLLPNVN
>Art1
YVDDLIFTGNNPSMFEEFKKEMTKEFEMTDIGLMSYYLGIEVKQEDNGIFITQEGYAKEVLKKFKMDDSNPVCTPMECGIKLSKKEEGE
GVDPTNLSLVGSLRYLTCTRPDILYAVGVVSRYMEHPTTTHFKAAKRILRYIKGTVNFGLHYSTTSDYKLVVCHAIWLRNLLKELSLPQ
EEPTKIFVDNKSAIALAKNPVFHDRSKHIDTRYHYIRECVSKKDVQLEYVKTHDQVADIFTKPLKREDFIKMRSLLGVAKSSLRGGVES
>BARE-1
YVDDILLIGNGVEFLENIKDYLNKSFSMKDLGEAAYILGIKIYRDRSRVIGLSQSTYLDKVLKRFKMEQSKKGLLPVLQGTRLSKTQCP
ATDKDIEHMSTVPYASAIGSIMYAMLCIRPDVSLAISMAGRFQSNPGVDHWMAVKNILKYLKRTTEMFLVYGGDKELAVKGYVDASFDT
DPDDSKSQTGYVFILNGGVVSWCSSKQSVVADSTCEAEYLAASEATKEGVWMKQLMTDLGVVSSALNPITLFCDNMGVIALAKEPQFHK
NTIRIKRRFNLIRDYVEEEDVNICKVHMDLNVAPAD
>Evelknievel
YVDDMLITGSDTNIITRFIANLAARFSLKDLGEMSYFLGIEATRTSKGLHLMQKRYVLDLLEKTNMLAAHPVLTPMSPTPKLSLTSGKP
LDKPSEYRAVLGSLQYLLFTRPDIAYAVNRLSQYMHCPTDLHWQAAKRILRYLAGTPSHGIFIRADTPLTLHAYSDADWAGDIDNYNST
NAYILYLGSNPISWSSKKQKGVARSSTEAEYRAVANATSEIRWVCSLLTELGITLSSPPVVYCDNVGATYLSANPVFDSRMKHIALDFH
FVRESVQAGALRVTHVSTKDQLADALTKPLPRQPFTTLISKIGVAKAPPS
>Mosqcopia
HVDDILVASAEEANVKREFENLGREFELTCLGEIRHFLGVEVLREDGVFKIRLKQFIDKLIIKHGMENAKTTRSPMDIGFLKDGANSEP
FEDVTLYRSLVGGMLYLSVVARPDIAASTAILGRKFSEPSQADWTAAKRLLRYLKATRHYFLRLGGAAEDPLVGYSDADWAGDPVSRRS
TSGFVFLFAGGTVSWASRRQTCVTLSSMEAEYVALAEACQETIWLRQLLRDFGEPQLQPTTMKEDNQGCIAFIKTERSSKRSKHINTKE
RFVQELCEKNEIVLEYCPTEIMIADVMTKPLGPQKHGDFVVKLGLEDPQ
>Osser_
YVDDLLIAAKDINIVRQLKDKLKSILMCVTWVRPVCFLGFEIERNRAERTMKVSQKRYAKGLVEKYGLEDANGRAVPLSHGTTLHSAGQ
PLDVRQFRFGELIGSLLYLSVGTRPDIAYSVGALARHMSKPTVEHWQAAKGVVRYIAGTAGLGIVFSPKSSNPSLHGYCDSDYAACVDT
RRSTTGYVFLNAGGAVSWSSRLQPTVATSTAEAEYMASGAATKEALWYRHLARDLQMRVSSVPILCDSQAAIKIINNPISSARSKHIDV
LHHFVRERVARGEIVFSYCKSADMVADCLTKPLPLPQFKKCIQAMGLR
>Panzee
YVDDMLVVGPNKDQIQELKAQLAEFDMKDLGPANKILGMQIHRDKKDRKIWLSQRNYLLKVLRRFNMQDCKPISTPLPVNYKLSSSMSP
SNEAERMEMSRVPYASAVGSLMYAMICTRPDIAQAVGAVSRFMADPGREHWSIVKRILRYIKGTSDVALCFEGSEFVVRGYVDSDFAGD
LDKRKSTTGYVFTLAGGAVSWLSKLQTVVALSTTEAEYMAATQACKEAIWIQRLLEELGHKQEKITVYCDSQSALHIARNPAFHSRTKH
IGIQYHFVREVVEEGKVDMQKIHTKDNIVDVMTKPVNTDKFTWCRSLFGLLKT
>Retrofit
YVDDIIVASSTEKATTALLKDLNKEFALKDLGDLHYFLGIEVTKVSNGVILTQEKYANDLLKRVNMSNCKPVSTPLSVSEKLTLYEGSP
LGPNDAIQYRSIVGALQYLTLTRPDIAYSVNKVCQFLHAPTTSHWIAVKRILRYLNQCTSLGLHIHKSASTLVHGYSDADWAGSIDDRK
STGGFAVFLGSNLVSWSARKQPTVSRSSTEAEYKAVANTTAELIWVQTLLKELGIESPKAAKIWCDNLGAKYLSANPVFHARTKHIEVD
YHFVRERVSQKLLEIDFVPSGDQVADGFTKALSACLLENFKHNLNLARL

```
>Ta1-3
YVDDMLIAGKSKSEINKVKEQLSMEFEMKDMGPASRILGIDIIRDMKNGVLRMSQASYIHNVVQRFNMAEAKVTRSPIGAHFKLAAVRD
DDECIDNNAVPYASAVGSIMYAMIGIRPDLAYVICLVSRYMARPGSIHWEAVKWILRYMRGSQDLNLVFTKEKEFRVTGYCDSDYAADL
DRRRSVSGYVFTVGGNTVSWKANLQSVTALSTTEAEFMALTEAAKEALWIKGLMKDLGLEQDKVTLWCDS*SAICLFKNSTHHERTKHI
DVRYNFIRDVVEAGDVDVLKIHTSRNPADALTKSIPVNKFQSALELLKLVKWD
>Tca5
YVDDILICAPSDSEIDQVKNNVRKYFSITDNALCRTFLAINVYHQANDIRLSLNDYIRRMIQELKLSVSETNPVSIPSDVNYEIFRVNE
NDDEKPCDQTKYRSLIGKLLFASNTIRFDIAYSVNSLSRFINDPKEKHWIAAVKVVKYLSGTQRYGICSYGNGDFNIYADSDWASTPSD
RQSITGYIVTYAGAPISWRSKKQNVIALSTTEAEFMALTESIKEALWLIYIFRDINVILKLPIVIYEDNLLCQKLLENPRFHNRTKHID
LKYKFTKDHIEAGTIKVESTNSADNLADMLTKPLPKIKFKHLRWLAGLRPLD
>Tgmr
YVDDIILAGDNLKSITHFTKFLDQTFSIKDLGILKFFLGLEVARSSHGIHLCQGKYALDILSDSGMLGSRPTSTPMDYSTRLSASMGTP
LSETSASSYR*LIDRLIYLTNTRPNITHAVQQLSQYMANPTSTHSQATF*ILRYLKGSPGSGIFFAAHSTLTLKAFSDSDWTGCRDTRC
SITGFSVYLGDYLISWRSKKQSTVSRSSSEAEYRALASTTCELQWLSYLLHDFWVPFLQPATLYCDNQSAIQIASNPVFHERTKHIEID
CHIVRDKVNAGLLKLLPVSSSMQLADIFTKPLTPAIFQGLCSKLGMMNIHSQL
>Ty1
FVDDMVLFSKNLNSNKRIIEKLKMQYDTKIINLGESDEEIQYDILGLEIKYQRGKYMKLGMENSLTEKIPKLNVPLNPKGRKLSAPGQP
GLYIDQDELEIDEDEYKEKVHEMQKLIGLASYVGYKFRFDLLYYINTLAQHILFPSRQVLDMTYELIQFMWDTRDKQLIWHKNKPTEPD
NKLVAISDASYGNQPYYKSQIGNIYLLNGKVIGGKSTKASLTCTSTTEAEIHAISESVPLLNNLSYLIQELNKKPIIKGLLTDSRSTIS
IIKSTNEEKFRNRFFGTKAMRLRDEVSGNNLYVYYIETKKNIADVMTKPLPIKTFKLLTNKWIH
>Ty4
YVDDCVIAASNEQRLDEFINKLKSNFELKITGTLIDDVLDTDILGMDLVYNKRLGTIDLTLKSFINRMDKKYNEELKKIRKSSIPHMST
YKIDPKKDVLQMSEEEFRQGVLKLQQLLGELNYVRHKCRYDIEFAVKKVARLVNYPHERVFYMIYKIIQYLVRYKDIGIHYDRDCNKDK
KVIAITDASVGSEYDAQSRIGVILWYGMNIFNVYSNKSTNRCVSSTEAELHAIYEGYRDSETLKVTLKELGEGDNNDIVMITDSKPAIQ
GLNRSYQQPKEKFTWIKTEIIKEKLKRSITVKITGKGNIADLLTNQYQHLILKDLYKY
>AtRe2
YVDDILITGNDTVLLKHTLDALSQRFSVKEHEDLHYFLGIEAKRVPQGLHLSQRRYTLDLLARTNMLTAKPVATPMATSPKLTLHSGTK
LPDPTEYRGIVGSLQYLAFTRPDLSYAVNRLSQYMHMPTDDHWNALKRVLRYLAGTPDHGIFLKKGNTLSLHAYSDADWAGDTDDYVST
NGYIVYLGHHPISWSSKKQKGVVRSSTEAEYRSVANTSSELQWICSLLTELGIQLSHPPVIYCDNVGATYLCANPVFHSRMKHIALDYH
FIRNQVQSGALRVVHVSTHDQLADTLTKPLSRVAFQNFSRKIGVIKVPPSCGG
>Wis2
YVDDILLMRNDIEFLESIKAYLNKCFSVKDLGEAAYILGIKIYRDRSRRLIGLSQSTYLDKILKKFNMDQSKKGFLPVLQGVQLSTAQC
PTTAEDREKMSVIPYASAIGSIMYAMLCTRPDVKLVVSLVGRYQSNPGMEHWTAVKNILKYLKRTKDMFLVYGGDEELVVKGYVDASFD
TDLDDSKSQTGYVYILNGGAVSWCSCKQSVVAGSTCEAEYMAASEAAQEAVWMKEFITDLGVIPNASGPMTLFCDNTGAIALAKEPRFH
RKTRHIKRRFTSIRESVQNGDIDICKVHTDLNVADPLTKPLPRAKHDQHQDAMGVRFITM
>Retrosor4
YVDDIIVASSSPDATKALLVDLQHDFALKDLGDLHYFLGIEVKRGNTSMLLTQERYATEVLKRSGMDKCHPVDTPLCSSEKLSIEDGNK
LGSEDATKYCSVVGALQYLTLTRPDISFAVNKVCQFLHSPTTIHWSAVKRILRYIKGTTKLGIKFSKSESMLVSAFADADWAGCVDDRR
STGGFAVFLGNNLISWTAKKQPTVSRSSTEAEYKALANATAEMIWVQKLLNELGVRHPPRARLWCDNLGATYLSANPIFHARTKHIEID
FHFVRERVAQKLLEVRFISTGDQLADGFTKALPVIKLKKFRDNLNLQSG
```
